# Connectivity within regions characterizes epilepsy duration and treatment outcome

**DOI:** 10.1002/hbm.25464

**Published:** 2021-05-11

**Authors:** Xue Chen, Yanjiang Wang, Sebastian J. Kopetzky, Markus Butz‐Ostendorf, Marcus Kaiser

**Affiliations:** ^1^ College of Control Science and Engineering China University of Petroleum (East China) Qingdao China; ^2^ School of Computing Newcastle University Newcastle upon Tyne UK; ^3^ Biomax Informatics AG, Brain Science Planegg Germany; ^4^ TUM School of Life Sciences Weihenstephan Technical University of Munich Freising Germany; ^5^ NIHR Nottingham Biomedical Research Centre, School of Medicine University of Nottingham Nottingham UK; ^6^ Sir Peter Mansfield Imaging Centre, School of Medicine University of Nottingham Nottingham UK; ^7^ School of Medicine Shanghai Jiao Tong University Shanghai China

**Keywords:** epilepsy duration, high‐resolution structural network, network metrics, surgical outcome prediction, within brain region

## Abstract

Finding clear connectome biomarkers for temporal lobe epilepsy (TLE) patients, in particular at early disease stages, remains a challenge. Currently, the whole‐brain structural connectomes are analyzed based on coarse parcellations (up to 1,000 nodes). However, such global parcellation‐based connectomes may be unsuitable for detecting more localized changes in patients. Here, we use a high‐resolution network (~50,000‐nodes overall) to identify changes at the local level (*within* brain regions) and test its relation with duration and surgical outcome. Patients with TLE (*n* = 33) and age‐, sex‐matched healthy subjects (*n* = 36) underwent high‐resolution (~50,000 nodes) structural network construction based on deterministic tracking of diffusion tensor imaging. Nodes were allocated to 68 cortical regions according to the Desikan–Killany atlas. The connectivity *within* regions was then used to predict surgical outcome. MRI processing, network reconstruction, and visualization of network changes were integrated into the NICARA (https://nicara.eu). Lower clustering coefficient and higher edge density were found for local connectivity *within* regions in patients, but were absent for the global network *between* regions (68 cortical regions). Local connectivity changes, in terms of the number of changed regions and the magnitude of changes, increased with disease duration. Local connectivity yielded a better surgical outcome prediction (Mean value: 95.39% accuracy, 92.76% sensitivity, and 100% specificity) than global connectivity. Connectivity *within* regions, compared to structural connectivity between brain regions, can be a more efficient biomarker for epilepsy assessment and surgery outcome prediction of medically intractable TLE.

## Graphical abstract

- Local structure within regions from high‐resolution network reveals epilepsy abnormality.
- The extent of within‐region abnormal changes increases for long‐term epilepsy and bad surgery outcome patients.
- Within‐region change can be a novel network biomarker that is better than current approaches.

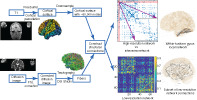

## 1 INTRODUCTION

Brain network disorders lead to changes in structural and functional connectivity between brain regions (Bonilha et al., 2012). These changes are supposed to result from changes within brain regions such as cell death or changes in synaptic connectivity (Jenner & Olanow, 1998; McGlashan & Hoffman, 2000). While cell death, unless being compensated by excess growth of other cell types, can be observed through changes in cortical thickness (Pereira et al., 2012), changes in local connectivity remain elusive. Using high‐resolution structural connectivity, where the cortical surface is parcellated into 50,000 regions of interest (ROIs) of comparable size, we test whether brain network disorders are visible at this local level, observing connectivity within brain regions, by looking at the case of epilepsy.

Temporal lobe epilepsy (TLE) as the most common type of epilepsy is characterized by recurrent seizures and can be controlled using antiepileptic medications or surgery (Wiebe, Blume, Girvin, & Eliasziw, 2001). For surgery, locating, and removing epileptogenic zones (EZ) which are involved in seizure generation is crucial; however, seizures can be caused by a complex network rather than a single region (Bartolomei, Chauvel, & Wendling, 2008). Structural connectivity between regions shows distinct changes for patients leading to changes in network dynamics (Hutchings et al., 2015). However, despite studies on cortical thickness (Pereira et al., 2012), surface area (SA) (Taylor et al., 2015), or gray/white matter volume (Beheshti et al., 2018; Bernasconi et al., 2004), there have been no studies investigating network changes within brain regions.

In this study, we constructed high‐resolution structural networks with around 50,000 nodes that form cortical regions to observe local connectivity within brain regions. We compared our results with a low‐resolution global network where 68 cortical regions formed the network nodes. High‐resolution networks with 1,000 or more nodes were proposed in the past (Besson et al., 2017; Irimia & Van Horn, 2016; Taylor, Wang, & Kaiser, 2017) and, using the parcellation into 50,000 nodes on healthy subjects, a modular organization within brain regions could be established (Taylor, Wang, & Kaiser, 2017).

Based on this approach, we investigated how epileptogenic networks change at the local level, within brain regions. Do these local changes also correlate with epilepsy duration (Besson et al., 2017)? Which cortical regions are most informative when predicting surgery outcome? And, most importantly, is the predictive power for surgery outcome based on high‐resolution local network measures more informative than that based on normal low‐resolution global networks?

## 2 METHODS

### 2.1 Subject information

Data were studied retrospectively for 33 patients with mesial TLE and 36 age‐ and sex‐matched healthy control subjects (Table 1). Diffusion tensor imaging (DTI) data (voxel size =2 × 2 × 2mm, TR = 10,000 ms, TE = 91 ms, FOV = 256 mm) with 64 diffusion directions (*b* = 1000s/mm^−2^ ) and 12 b0 images were obtained. T1‐weighted MRI scans used 1 mm isovoxel, FOV = 256 mm, TR = 2,500 ms and TE = 3.5 ms. All patients underwent a comprehensive pre‐surgical evaluation, and all had a confident diagnosis of mesial TLE based on semiological, electrophysiological and imaging investigations. Amygdalohippocampectomy and hippocampalsclerosis were confirmed histologically using standard criteria (Kreilkamp, Weber, Richardson, & Keller, 2017). No patients had undergone any previous neurosurgery before mesial TLE surgery. Informed written consent was signed for all participants. Disease duration was computed from age at first seizure onset to age at scan time. For surgery outcome, measured at least 1 year of postsurgical follow‐up, patients were classified using the International League Against Epilepsy (ILAE) surgical outcome scale.

**TABLE 1:** Demographics of study participants and the difference between patients and controls

	Num.	Gender male/female	Age [years] m/S.D.	Duration [years] m/S.D.	Surgical outcome ILAE 1–2/ ILAE 3–5
Con	36	17/19	39.06/12.32	–	–
TLE	33	15/18	38.79/12.79	23.45/15.03	21/12
LTLE	19	7/12	39.42/11.68	24.21/14.19	12/7
RTLE	14	8/6	37.93/14.57	22.43/16.60	9/5
Con vs TLE	–	*χ* ^2^ = 0.022; *p* = 0.883	*p* = 0.923	–	–
Con versus LTLE	–	*χ* ^2^ = 0.545; *p* = 0.460	*p* = 0.903	–	–
Con versus RTLE	–	*χ* ^2^ = 0.397; *p* = 0.529	*p* = 0.772	–	–
LTLE versus RTLE	–	*χ* ^2^ = 0.134; *p* = 0.247	*p* = 0.734	*p* = 0.744	*χ* ^2^ = 0.004; *p* = 0.947

*Note*: Con: healthy controls; TLE: all patients; LTLE/RTLE: patients who had left/right‐side surgery; m and S.D. represent the mean value and the standard deviation of each group, respectively. Chi‐square test was used to check for group differences in gender and surgical outcome. 5,000 permutation test was applied for age comparison. Bonferroni correction was performed due to the multiple comparisons. There is no difference in age, gender, duration, and surgical outcome between groups.

### 2.2 Data pre‐processing and structural network reconstruction

T1 images were pre‐processed with FreeSurfer (http://surfer.nmr.mgh.harvard.edu). Briefly, the pre‐processing steps included intensity normalization, skull stripping, and brain tissue segmentation. Cortical surfaces, such as the pial surface, were extracted and manually checked, edited so that its boundary did not cross the white matter surface boundary. All defects arising from this procedure were manually corrected. We down‐sampled the pial surface files with an output ratio of 0.1 using the Matlab toolbox “IsoMesh” (Fang & Boas, 2009) and constructed structural connectomes (Taylor, Wang, & Kaiser, 2017). The final surfaces were composed of ~50,000 triangles, each representing a node in the network and with an average SA of approximately 3.5 mm^2^.

**FIGURE 1:**
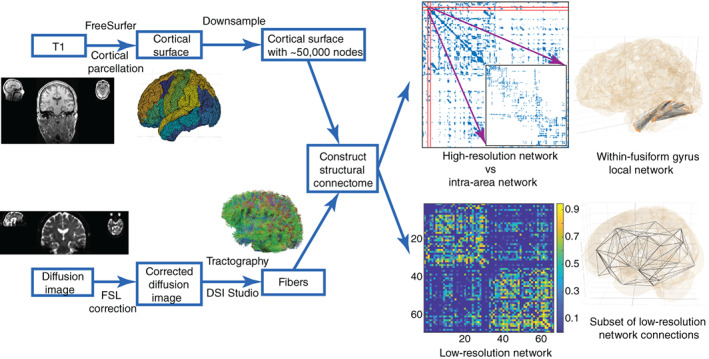
Constructing global (between‐area) and local (within‐area) structural networks. The final high‐resolution connectivity is composed of around 50,000 nodes. Zooming into the high‐resolution networks, each within‐area structure, for example within the fusiform gyrus (inset), can be considered as a small local network. Note that the local network contained connections among nodes within regions, but contained no connections between regions. The connectivity and 3D visual graphs, generated by NICARA, of an intra‐area network, for example, within‐fusiform gyrus network are shown here. The low‐resolution global connectivity has 68 nodes, each corresponding to a DK atlas area. The 3D subset of low‐resolution network connections is also shown by NICARA (in the right of low‐resolution network)

We used “DSI Studio” (http://dsi-studio.labsolver.org) to get deterministic streamline tractography from eddy current‐corrected diffusion tensor images which have been processed in FSL (https://fsl.fmrib.ox.ac.uk/fsl/fslwiki/). Generalized q‐sample imaging (GQI) method with diffusion sampling length ratio of 1.25, 8‐fold orientation distribution function (ODF) tessellation with five peaks resolved on an ODF was chosen when reconstructing FIB files. Directions at corpus callosum were checked to look well (i.e., clean fiber direction at mid corpus callosum and crossing patterns at lateral corpus callosum). Whole‐brain seeding, 0.6*(Otsu's threshold) quantitative anisotropy level, 60 turning angle threshold, 10 mm < tracts length < 300 mm were set and a total of 10,000,000 streamlines was saved. Surface files, streamlines, and “aparc+aseg.nii” files generated by FreeSurfer were linearly registered into the same space. Streamlines whose endpoints terminating within the gray matter were acquired. The high‐resolution connectivity matrix was defined by streamline counts between the centers of the closet triangles (Euclidean distance) and was normalized by triangle area. Nodes were sorted according to the default FreeSurfer parcellation, the Desikan–Killany (DK) atlas. Each cortical DK area included hundreds of nodes and can shape an intra‐area network. We counted inter‐area streamlines and normalized with a logistic function to create weighted low‐resolution networks (68 cortical areas) (Hutchings et al., 2015). Refer to Figure 1 and Supplementary Text S1 in Supplementary Material for more details. To allow for a combination of left and right TLE and investigate ipsi‐ and contra‐lateral differences, the right regions were flipped in patients who had a right‐side surgery (Mueller et al., 2009; Taylor et al., 2018). The demographics of 14 right TLE patients were shown in Table 1. The same was done with control images. Supplementary Material Table S1 lists the abbreviations of all cortical DK areas. We use the term “area” or “region” to refer to a FreeSurfer (DK) area in the following.

To handle the massive amount of connectivity data that is generated by our within region connectivity workflow, we used NICARA (https://nicara.eu) (Kopetzky & Butz‐Ostendorf, 2018) – a novel software solution to manage large connectome data sets. We configured the above‐described imaging workflow in NICARA so that all external pre‐ and processing steps could be executed via the NICARA web portal. The resulting brain connectivity was automatically imported, partially analyzed and visualized by the NICARA.

### 2.3 Network analysis

TLE is often accompanied by surface‐based morphometry changes, such as SA reduction in the whole or parts of the brain (Taylor et al., 2015). Beyond surface abnormalities, structural topological organizations *between* regions were significantly different in patients. To investigate and elucidate epileptic brain pathology *within* and *between* cortical areas, nine metrics including SA before down‐sampling, average fiber length (FL), connectivity strength (S), and six topological network properties (Kaiser, 2011; Rubinov & Sporns, 2010) were considered. For the low‐resolution network of connections between 68 regions, we used nodal connectivity strength (*S*_i_), efficiency (*E*_i_), and clustering coefficient (*C*_i_). Average FL was the sum of all of the streamline trajectory lengths divided by the total number of streamlines. Connectivity strength was the sum of connection weights which were normalized by triangle SA for high‐resolution networks (Besson, Lopes et al., 2014) and by a logistic function for low‐resolution networks (Hutchings et al., 2015). As the high‐resolution network is very sparse with about 50,000 nodes and fewer streamlines between nodes, we used binary (unweighted) connectivity to calculate network properties. On contrary, the denser low‐resolution network with 68 nodes was weighted in line with previous studies (Bonilha et al., 2015; Hutchings et al., 2015).

Concerning six topological network features, edge density (*d*) represents the ratio of present connections relative to the number of possible connections. Characteristic path length (*L*) is the average number of connections within the shortest path from one node to another. Global efficiency (*E*_global_) is a measure of the shortest paths in the network. The average clustering coefficient (*C*) measures how well neighbors of a node are connected. Local efficiency (*E*_local_) is an alternative measure of connectivity between neighbors. As the previous study mentioned, above properties are always sensitive to the sparsity of the network (Van Wijk, Stam, & Daffertshofer, 2010). For our sparse high‐resolution network, we therefore normalized by network properties of 1,000 randomly rewired networks with the same degree distribution. We also normalized low‐resolution network measures by values from 100 rewired networks with the same degree and strength distribution. Finally, we calculated small‐worldness (*σ*). Brain Connectivity Toolbox was used to compute network properties (Rubinov & Sporns, 2010). All measures were corrected for age and gender using a general linear regression model generated from healthy subjects. The relative definitions of all above metrics were listed in Supplementary Material Text S1.

### 2.4 Within‐area local organization analysis

Zooming into high‐resolution networks, each DK cortical area can be regarded as a relatively small subnetwork that has 100–1,500 nodes. Regardless of inter‐area interactions, the SA of each DK cortical regions was first examined. As the morphometry variation may lead to changes in connectivity, we then regressed out the SA effect by a general linear regression model to pinpoint the influence of other metrics, especially for topological properties (see Supplementary Material Figure S1). Network size (i.e., the number of nodes) is another potentially confounding factor: comparisons between networks with different sizes can yield spurious results (Van Wijk, Stam, & Daffertshofer, 2010). We, therefore, checked network size effects using Spearman's rank correlation on metrics of all controls that included within‐area networks of variable size (see Supplementary Material Figures S2 and S3). In the following analysis, we excluded features that showed significant changes in network size.

### 2.5 Epilepsy duration and surgical outcome analysis

Brain features were proved to change with the duration of epilepsy in patients. Gray matter concentration (gray matter volume divided by total intracranial volume) had a negative association with the duration of epilepsy within the hippocampus, temporal lobe and several limbic structures (Bonilha et al., 2006). Hippocampal volume reduced more if patients had a long history of epilepsy before surgery (Theodore et al., 1999). The alterations of cortical thickness correlation networks intensify over time (Bernhardt et al., 2011). To study epilepsy duration effects, we grouped epilepsy patients into two categories (fewer and more than 20‐year duration). At the threshold of 20‐year disease duration, two groups had matched group size, gender, surgical outcome, and surgery side distribution, which was beneficial for the following group comparison. For comparison, controls were also categorized into two groups whose age and gender were matched with patient groups. Two control groups were maximum overlapped so that patients can be compared to the same healthy controls. Shorter epilepsy duration is demonstrated to make good surgery outcomes more likely (Bjellvi, Olsson, Malmgren, & Ramsay, 2019). We therefore also examined network differences between patients with surgical outcome. Patients here were classified into two groups: good postsurgical outcome (class ILAE 1–2, 21 patients) and bad (class ILAE 3–5, 12 patients) postsurgical outcome. Refer to Supplementary Material Figure S4, Table S2 for more details.

### 2.6 Statistical analysis

Network metrics comparison used a two‐sided 5,000 permutation test (Zhang et al., 2011) with a statistical significance set as *p* < 0.05. Cohen's *d* measures difference size between two means with effect size “huge,” “very large,” “large,” “medium,” “small,” “very small” set correspond to thresholds of *d* = 2.0, *d* = 1.2, *d* = 0.8, *d* = 0.5, *d* = 0.2, *d* = 0.01, respectively. To balance left/right TLE effects and make results more reliable, the Cohen's *d* values of group comparisons were weighted by the sample sizes of left/right TLE patients (Borenstein, Hedges, Higgins, & Rothstein, 2009). The weighted Cohen's *d* was defined by $\frac{n_{1}*d_{1}+n_{2}*d_{2}}{n_{1}+n_{2}}$, where n_1_/n_2_ was the sample size of left/right patients; *d*1/*d*2 represented the group comparison effect size between left/right TLE patients and control subjects. Spearman's rank correlation and Pearson's linear correlation were calculated to study changes related to epilepsy duration. The absolute *z*‐scores of patients, showing the deviation of measures from the control group, were then tested to predict surgical outcome. Bonferroni correction was performed, since multiple comparisons were analyzed.

### 2.7 Outcome prediction analysis

Classification learner, a component of MATLAB, was applied to test the outcome predictive ability of network metrics. For surgical outcome prediction, only one feature was tested at once for classification and every measure including average FL, connectivity strength, and six topological network properties in each local region was checked to get measures with good prediction performance. Training models were selected among six classical methods: tree (TR), discriminant (DM), logistic regression (LR), support vector machines (SVM), k‐nearest neighbor (KNN), and ensemble (ENS). To avoid overfitting, 5‐fold cross‐validation was performed by partitioning the data set into folds and estimating accuracy on each fold. The same procedure was repeated and the average estimation was taken over 50 times. The optimal point of receiver operating characteristic (ROC) curve was used as a threshold for classification and was shown as the best prediction point. To explore how metrics make the good and poor surgical outcome, 5,000 permutation tests and Cohen's *d*‐score were considered.

## 3 RESULTS

### 3.1 Changes in whole high‐ and low‐resolution networks

In line with previous studies (Taylor et al., 2015), the whole brain SA was reduced in patients compared to controls (Supplementary Material Figure S5). The majority of cortical regions, in particular around the ipsi‐lateral temporal and frontal lobe, had significantly decreased SA in patients. Unlike the low‐resolution 68 region networks, the high‐resolution networks with around 50,000 nodes showed distinct higher connectivity strength, edge density, and lower global clustering coefficient. Both low‐ and high‐resolution networks observed a decreased average FL (*between*‐nodes) in patients.

### 3.2 Within‐region local changes identification

**FIGURE 2:**
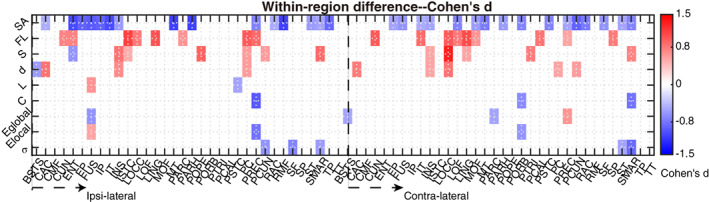
Identification of changed within‐area local networks. All Metrics of intra‐area networks shown in color are significantly different (*p* < 0.05) in patients with lower values for negative Cohen's *d* (blue) and higher values for positive Cohen's *d* (red) in patients compared to controls. Several regions, both ipsi‐ and contra‐lateral to the epileptic focus, showed changes in one or more local network features. Network features were surface area (SA), fiber length (FL), total connectivity strength (*S*), edge density (*d*), characteristic path length (*L*), clustering coefficient (*C*), global efficiency (*E*_global_), average local efficiency (*E*_local_), and small‐worldness (𝜎)

The changed metrics were plotted in Figure 2 across all intra‐area networks after network size effects were eliminated. The absolute Cohen's *d* values of all metrics were summed for each region to measure how different the areas are in patients (Supplementary Material Table S3). Ipsi‐lateral precentral gyrus (PREC) and contra‐lateral supra marginal (SMAR) showed great changes with both decreased clustering coefficient. Moreover, contra‐lateral SMAR, lateral occipital (LOCC), pars triangularis (PTRI), and ipsi‐lateral insula (INS), SMAR, pars opercularis (POPE) showed increased connectivity strength. About half of the affected regions were located in the temporal and frontal lobe. The increase of average FL *within*‐region was dramatic and widespread. However, the average FL across the whole brain was reduced (Supplementary Material Figure S5) which suggests the loss of long‐length fibers *between* regions in patients.

**FIGURE 3:**
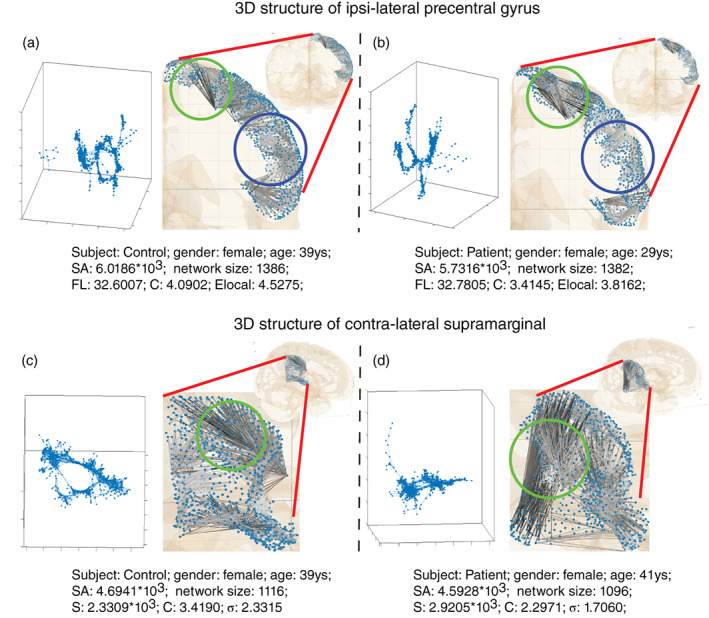
Visualization of local network changes as exemplified for the ipsi‐lateral precentral and contra‐lateral supra marginal region. From left to right, two subjects (one control, one patient) are shown for both ipsi‐lateral precentral gyrus ((a) and (b)) and contra‐lateral supra marginal ((c) and (d)). While the network size (the number of nodes) and surface area size [mm^2^] (SA) remain comparable, clustering coefficient (c), local efficiency (Elocal) are reduced in ipsi‐lateral precentral gyrus (the upper panel: (a) and (b)) for patients. And reduced clustering coefficient, small‐worldness (*σ*), and increased connectivity strength (*S*) are found in contra‐lateral supra marginal for patients (the lower panel: (c) and (d)). Local networks were visualized by two methods: force‐directed layout in the left of all sub‐figures and anatomical layout by the NICARA in the right of all sub‐figures. Compared with the control (a), the patient (b) had more long connections in the precentral gyrus marked by green circles and fewer short connections in the blue‐circle zones. Similarly, compared with the control (c), the patient (d) had more remote long connections in the zone marked by blue circles. Anatomical layout connections were plotted in white to dark color to represent short to long fibers in figures

To show examples for within‐region local changes, we select two regions which have the most changes in patients compared with controls (cf. Supplementary Material Table S3): the ipsi‐lateral PREC and the contra‐lateral SMAR (See Figure 3). Compared to controls with a matched number of nodes, the modular organization was reduced with lower clustering coefficient (*C*) and local efficiency (*E*_local_) in ipsilateral PREC, specifically given that more local neighbor nodes were disconnected (e.g., the network in the left panel of Figure 3(b) is less locally connected with several separate branches and isolated small cliques compared with that in Figure 3(a)). Looking at the connected networks in the brain (i.e., the right panel of Figure 3(a) and (b)), we observed that, unlike in the control, the local short connections in light‐gray color were fewer in the lateral PREC gyrus marked by a blue circle. That may cause a reduced clustering coefficient and local efficiency. While the superior PREC gyrus marked by green circles showed more long connections in dark color. Similarly, even though there were more connections of contra‐lateral SMAR in patients (see increased connectivity strength (*S*) in Figures 2 and 3(d)), the more remote long connections (i.e., zones marked by blue circles) may make *within*‐region networks less localized leading to lower clustering coefficient and small‐worldness (*σ*).

### 3.3 Duration effects for epileptogenic intensity

**FIGURE 4:**
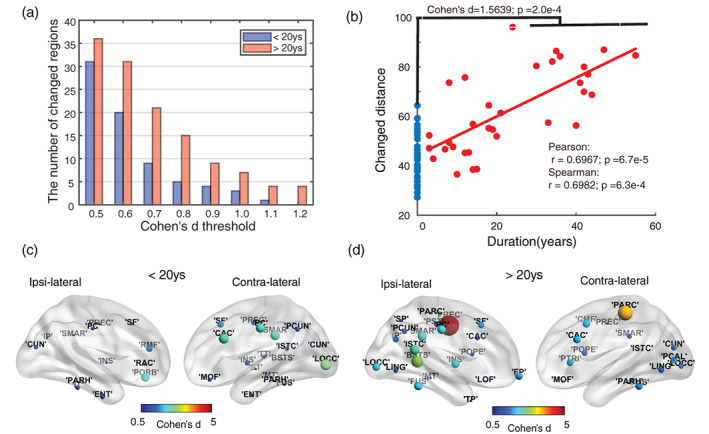
The intra‐regional change patterns related to duration. Patients were grouped into two parts: one whose epilepsy duration is smaller than 20 years, the others above. (a) The number of abnormal cortical areas among 68 regions increased with duration when changed regions were thresholded through medium (Cohen's *d* > 0.5) to very large (Cohen's *d* > 1.2) changes in network features. (b) Changed distance was computed by summing up absolute *z*‐scores across all metrics and for all duration‐related regions. Blue dots show the distribution of self‐control distance. Red dots represent the patient‐control distance showing that the intensity increases with disease duration. Furthermore, using a permutation test, there was a wider range of distances to the average control group value for epilepsy patients than for controls (Cohen's *d* = 1.5639). (c) and (d) depict changed regions (*p* < 0.05, Cohen's *d* threshold = 0.5) for both durations. The color shows the sum of Cohen's d scores across all significantly changed metrics of a region. For the longer duration (> 20 years), changes get stronger especially in the ipsi‐lateral hemisphere and more largely changed regions are around ipsi‐lateral cingulate regions

Although the local network architecture is widely changed for patients, the number of changed network features and the extent of their alteration, relative to controls, vary between regions. Indeed, more regions are affected in patients with longer epilepsy duration (>20 years; Figure 4(a)). By summing up *z*‐scores across network features for regions related to duration (Supplementary Material Table S4) to give a distance from controls, we see a positive relationship (Figure. 4(b)) between alteration intensity and epilepsy duration (Pearson's correlation coefficient: 0.6652, Spearman's rank coefficient: 0.6887). Moreover, the patient‐control distance is significantly larger than the within‐control distance (5,000 permutation tests, *p* < 0.001, Cohen^′^s *d* = 1.5369). Compared to shorter duration, for longer duration more ipsilateral regions were affected beyond the temporal‐frontal regions (Cohen's *d* > 0.5; Figure 4(c), (d)). The abnormality for longer duration is more obvious in the ipsi‐lateral hemisphere compared with the contra‐lateral hemisphere. Regions that change the most for patients with more than 20‐year epilepsy were mostly distributed around the ipsi‐lateral PREC and cingulate (see Figure. 4(d)), such as the banks of the superior temporal sulcus (BSTS), the isthmus of the cingulate (ISTC), and the posterior cingulate (PC). Ipsi‐lateral caudal anterior cingulate (CAC) and contra‐lateral pericalcarine (PCAL) are highly related to duration (Supplementary Material Table S4). Notably, according to the mapping between DK regions and functional networks (Kabbara et al., 2017), precuneus (PCUN), lateral orbitofrontal (LOF) cortex, which are part of default‐mode network (DMN) and CAC, POPE, pars orbitalis (PORB) that are in the dorsal attentional network (DAN) show a positive duration relation.

### 3.4 Surgical outcome difference

**FIGURE 5:**
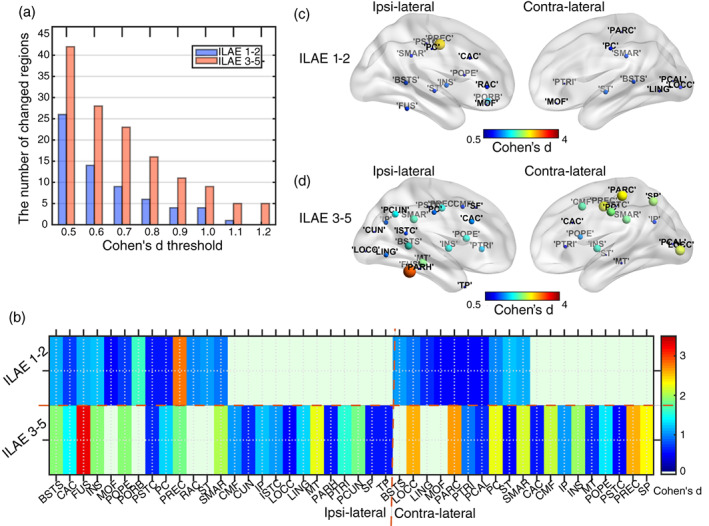
Local network abnormalities related to surgical outcome. Patients were categorized into two groups: good surgery outcome (ILAE 1–2) and bad surgery outcome (ILAE 3–5). (a) The number of abnormal cortical areas, for medium (Cohen's *d* > 0.5) and very large (Cohen's *d* > 1.2) changes in network features, was higher for bad outcome patients. (b) Abnormal regions were further grouped into ipsi‐ and contra‐lateral regions relative to the side of surgery. The color shows the sum of Cohen's *d* scores across all significantly changed (*p* < 0.05) metrics of a region. For bad‐outcome patients, more regions are affected and changes are more pronounced (higher Cohen's *d*). (c, d) Changed regions (*p* < 0.05, Cohen's *d* threshold = 0.5) for both outcome groups. The color shows the sum of Cohen's *d* scores across all significantly changed metrics of a region. For the bad surgery outcome patients (ILAE 3–5), changes are stronger in both ipsi‐ and contra‐lateral hemispheres and more areas are involved

Does the extent of local network changes also indicate surgery success? Indeed, patients with bad outcome show more abnormal regions (Figure 5(a)). By summing up all Cohen's *d*‐score for locally changed network features (Figure 5(b)) to estimate abnormality, regions such as the ipsi‐lateral BSTS and insula (INS) were clearly changed for both good‐ and bad‐outcome patients but differences were stronger for bad‐outcome patients. However, regions such as contra‐lateral SMAR, CAC, caudal middle frontal (CMF) show abnormality for bad‐outcome but not for good‐outcome patients. Overall, bad surgical outcome was related to more regions showing strong changes, in particular ipsi‐lateral temporal‐cingulate‐frontal regions and contra‐lateral frontal–parietal brain regions (Figure 5(c), (d)). We also found abnormal regions were more obvious and widespread in bad‐outcome patients and even had larger abnormalities in regions of the contra‐lateral hemisphere.

### 3.5 Prediction of surgical outcome

Can the network changes be biomarkers for predicting surgery outcome? For the *within*‐area network shown in Table 2, ipsi‐laterally, for example, the metrics of BSTS (80.31±5.39% accuracy), the cuneus (CUN, 77.37±2.35% accuracy), INS (74.05±1.91% accuracy), pars triangularis (PTRI, 75.40±1.96% accuracy), and the superior temporal (ST, 73.23±1.24%) were tested as good predictors of surgery outcome. Contra‐laterally, the metrics of CAC (accuracy: 81.20±2.37%), CMF (77.80±2.12% accuracy), LOF (78.50±2.98% accuracy), PORB (74.65±1.86% accuracy), SMAR (74.41±2.14% accuracy), and temporal pole (80.40±3.67% accuracy) were successful predictors as well. Combining *within*‐region information, the predictive power results in a remarkable 0.93±0.01 AUC (Area under the ROC curve), 93.81±0.60% accuracy, 90.38±0.67% sensitivity, and 99.83±1.18% specificity. If considering SA effect (Supplementary Material Table S5), the prediction performance improves to an 0.97±0.02 AUC, 95.39±1.86% accuracy, 92.76±2.92% sensitivity, and 100±0.00% specificity.

**TABLE 2:** Predictions of surgical outcome for *within*‐region metrics

Area	Metric	AUC	Acc.(%)	Sen.(%)	Spec.(%)	Model	FN
(I)BSTS	*L*↑	0.74±0.02	74.24±1.87	80.42±4.28	63.43±6.48	ENS	–
(I)BSTS	*E*_local_↑	0.79±0.05	80.31±5.39	85.20±5.59	71.60±8.93	TR	–
(I)CUN	*L*	0.72±0.01	77.37±2.35	89.84±3.48	55.56±4.00	SVM	–
(I)CUN	*E*_global_	0.77±0.04	73.33±1.94	84.20±5.43	54.20±8.12	ENS	–
(I)INS	S↑	0.79±0.03	74.05±1.91	80.65±4.42	62.50±7.45	TR	SAN
(I)PTRI	*σ*↓	0.74±0.04	75.40±1.96	84.78±3.11	58.22±6.02	SVM	DAN
(I)PTRI	FL↓	0.73±0.02	73.11±1.07	88.10±2.55	46.88±4.31	TR	DAN
(I)ST	*C*↓	0.72±0.01	73.23±1.24	83.33±2.61	55.56±4.30	SVM	–
(C)CAC	*σ*	0.82±0.01	81.20±2.37	83.00±2.58	77.91±4.04	SVM	DAN
(C)CMF	*S*	0.81±0.02	77.80±2.12	78.78±4.41	76.00±7.68	ENS	SAN
(C)LOF	*C*	0.78±0.03	78.50±2.98	82.90±3.25	71.80±4.13	ENS	DMN
(C)LING	*σ*	0.73±0.02	76.74±1.93	96.50±3.17	42.16±2.00	SVM	–
(C)PORB	*L*	0.72±0.01	74.65±1.86	88.57±2.68	50.28±5.12	ENS	DAN
(C)PSTC	*L*	0.73±0.01	73.43±1.33	94.51±1.79	36.54±4.22	KNN	–
(C)PSTC	*E*_global_	0.74±0.02	73.81±1.51	90.48±0.00	44.64±4.44	SVM	–
(C)SMAR	*C*↓	0.73±0.02	74.41±2.14	78.04±4.36	68.06±4.29	ENS	SAN
(C)TP	*L*	0.86±0.04	80.40±3.67	77.45±3.47	85.54±9.28	TR	–
(C)TT	*E*_global_↑	0.75±0.04	73.83±1.53	81.82±4.16	59.85±8.18	TR	–
ALL	**–**	**0.93** **±** **0.01**	**93.81** **±** **0.60**	**90.38** **±** **0.67**	**99.83** **±** **1.18**	**DM**	
ALL+SA	**–**	**0.97** **±** **0.02**	**95.39** **±** **1.86**	**92.76** **±** **2.92**	**100** **±** **0.00**	**DM**	

*Note*: (I) and (C) represent ipsi‐lateral and contra‐lateral areas, respectively. 50 repetitions were performed to calculate the mean value and standard deviation. The metrics marked with ↓(↑) were significantly smaller (larger) in poor‐surgery patients (*p* < 0.01) compared with good‐surgery patients. Others without marks only show the good classification of *z*‐score but a slight difference between two surgical outcome groups. 5,000 permutation tests were used in predictions. The prediction performance of one metric is significant when *p* < 0.05 and metrics with good predictions were shown in the table. Abbreviations: AUC: area under ROC curve; Acc.: accuracy; Sen.: sensitivity; Spec.: specificity; DMN: default mode network; DAN: dorsal attentional network: SAN: salience network. Three networks are mentioned as some areas are part of specific functional networks. Predictive model types: Tree: TR; Discriminant: DM; Logistic Regression: LR: Support Vector Machines: SVM; K‐Nearest Neighbor: KNN; Ensemble: ENS. The best predictive models were shown in the table. ALL: means using the summed absolute *z*‐score values of all above measures to predict surgical outcome. ALL+SA: means using all predictors shown in Table 2 and SA metrics shown in Supplementary Material Table S5 to predict surgical outcome.

How does this performance compare to the standard approach of observing changes in the connectivity *between* regions, that is, our low‐resolution network with 68 cortical regions? For the low‐resolution network, the clustering coefficient of the ipsi‐lateral INS predicts outcome well with 75.76±4.29% accuracy performance (AUC: 0.74±0.05) which is similar to the 74.05±1.91% accuracy (AUC: 0.79±0.03) we get *for the same region* based on intra‐regional local connectivity. Moreover, across all regions, the top prediction is 0.76±0.02 AUC, 81.58±1.03% accuracy for inter‐area global network metrics (Supplementary Material Table S5) and 0.82±0.01 AUC, 81.20±2.37% accuracy for intra‐area local metrics (Table 2). Combining information from all low‐resolution network regions and including SA effects, performance improves (top: 0.95±0.01 AUC, 91.45±2.43% accuracy, 93.05±2.76% sensitivity, 88.67±4.38% specificity). However, performance for high‐resolution local network (0.97±0.02 AUC, 95.39±1.86% accuracy) is better than for the low‐resolution network. Predicted surgical outcomes for 33 patients by applying methods based on low‐ and high‐resolution networks are summarized in Table 3. About 93.81% of patients were predicted to be the same as the actual surgical outcome with about 90.38% sensitivity and 99.83% specificity for the high‐resolution network versus ~81.33% accuracy, 80.67% sensitivity and 82.50% specificity for the low‐resolution network. The major difference between high‐ and low‐ resolution methods was that the outcome prediction, in particular correctly identifying bad surgery outcomes, was much better for the high‐resolution within‐region network approach.

**TABLE 3:** Confusion matrix indicating the performance of high‐ and low‐resolution network in predicting surgical outcome

Network	Predicted outcome	Actual surgical outcome	
		Good = 21	Bad = 12
HighRes (SA out)	Good = 19.00±0.20 Bad = 14.00±0.20 Accuracy = 93.81±0.60 (%)	True positive = 18.98±0.14 False negative = 2.02±0.14 True positive rate, or sensitivity = 90.38±0.67 (%) False negative rate, or miss rate = 9.62±0.67 (%)	False positive = 0.02±0.14 True negative = 11.98±0.14 False positive rate, or fall‐out = 0.17±1.18 (%) True negative rate, or specificity = 99.83±1.18 (%)
LowRes (SA out)	Good = 19.04±0.35 Bad = 13.96±0.35 Accuracy = 81.33±1.28 (%)	True positive = 16.94±2.40 False negative = 4.06±2.40 True positive rate, or sensitivity = 80.67±1.14 (%) False negative rate, or miss rate = 19.33±1.14 (%)	False positive = 2.10±0.30 True negative = 9.9±0.30 False positive rate, or fall‐out = 17.50±2.53 (%) True negative rate, or specificity = 82.50±2.53 (%)
HighRes (SA in)	Good = 19.48±0.61 Bad = 13.52±0.61 Accuracy = 95.39±1.86 (%)	True positive = 19.48±0.61 False negative = 1.52±0.61 True positive rate, or sensitivity = 92.76±2.92 (%) False negative rate, or miss rate = 7.24±2.92 (%)	False positive = 0.00±0.00 True negative = 12.00±0.00 False positive rate, or fall‐out =0.00±0.00 (%) True negative rate, or specificity = 100±0.00 (%)
LowRes (SA in)	Good = 20.9±0.76 Bad = 12.1±0.76 Accuracy = 91.45±2.43 (%)	True positive = 19.54±0.58 False negative = 1.46±0.58 True positive rate, or sensitivity = 93.05±2.76 (%) False negative rate, or miss rate = 6.95±2.76 (%)	False positive = 1.36±0.53 True negative = 10.64±0.53 False positive rate, or fall‐out = 11.33±4.38 (%) True negative rate, or specificity = 88.67±4.38 (%)

*Note*: The predictors here were combined predictors that used the summed absolute *z*‐score values of all measures shown in Table 2 or Supplementary Table S5 to predict surgical outcome. 50 repetitions were performed to calculate the mean value and standard deviation. HighRes (LowRes): the overall prediction power combined with all high‐resolution/local predictors (low‐resolution/global predictors). SA in (SA out): the overall prediction power considering (not considering) SA predictors using high‐(HighRes) and low‐resolution (LowRes) method.

## 4 DISCUSSION

By using structural connectivity *within* and *between* cortical areas, based on DTI, we found significant alterations for TLE that were related to disease duration and surgery outcome. Different from previous epilepsy structural connectivity studies that focused on low‐resolution networks between brain regions (Taylor et al., 2018; Zhang et al., 2011), we focused on higher‐resolution structural networks with about 50,000 nodes for the first time, which showed more obvious topological changes than low‐resolution networks. For the local network *within* regions, significant changes in patients occurred, in particular for the ipsi‐lateral PREC and contra‐lateral SMAR. Moreover, the number of abnormal regions and the magnitude of network changes increased with disease duration in patients. While we have no longitudinal data, the link between local changes and disease duration suggests that *within*‐area connectivity could be a biomarker of TLE, potentially even before the onset of seizures. Finally, local changes *within* regions were successful predictors of surgery outcome (~95.39%/93.81% accuracy with/without SA effect). Predictive performance was better than for the standard approach of observing global changes in connections *between* regions indicating that *within*‐area connectivity can be useful in the future for planning surgical interventions.

### 4.1 Relationships to epileptic seizure pathways

Some regions with changes in local connectivity overlapped with seizure spreading pathways. A very common path for seizure spreading (Lieb, Dasheiff, & Engel, 1991) originates in the ipsi‐lateral temporal lobe and spread to the ipsi‐lateral frontal lobe before affecting the contra‐lateral frontal and temporal lobe. In our study, about half of the changed regions were located in the temporal and frontal lobe which could result from changes along activity propagation pathways. For example, the ipsi‐lateral entorhinal cortex (ENT), which participates in seizure generation and propagation, showed localized FL increase and decreased connectivity strength. The insular cortex as on the spreading pathways (Isnard et al., 2000) was observed a dramatically increased connectivity strength and higher edge density, which could be the reason for bad surgery outcomes when the insula was not considered as a surgery target (Harroud, Bouthillier, Weil, & Nguyen, 2012; Isnard et al., 2000)^.^ Besides, its higher connectivity strength was a good outcome predictor (~74.05% accuracy in Table 2). The locally changed subtemporal cortex, such as fusiform (FUS, decreased global efficiency) and parahippocampal gyrus (PARH, reduced SA), is also known to be involved in seizures (Alarcon et al., 1997). Changed regions beyond seizure propagation pathways might be indirectly affected by a region that is involved in seizure spreading.

### 4.2 Changes in TLE and their relation to function

Local network changes might also relate to functional changes in brain connectivity for epilepsy patients. For example, we see network changes in the primary motor cortex, the PREC gyrus, in line with previously reported ictal hyperperfusion due to seizure discharges (Tae et al., 2005). And pathological structural changes occur in PREC gyrus with smaller clustering coefficient and local efficiency indicating destroyed local interaction and lower stability to resist external interaction from other regions, which may reveal motor symptoms during epileptic seizures. Unfortunately, we cannot investigate whether the lost local connectivity involves excitatory or inhibitory (e.g., lateral inhibition) circuits. The loss of short‐length fibers *within*‐regions may cause a reduced local cluster organization. Fewer short‐length connections among some local nodes were observed in patients with locally increased mean FL. The decreased global mean FL *between* regions across the whole brain (Supplementary Material Figure S5(A)), however, indicates a loss of long‐length connections or more short‐range connections *between* regions in patients. It is consistent with the study of structure–function coupling during epileptic seizures (Shah et al., 2018) that demonstrated seizures may mainly rely on the underlying short‐range structural connections to propagate, in terms of both fiber trajectory length and Euclidean distance. The SMAR gyrus, a portion of the parietal lobe of the brain, plays a role in phonological processing (i.e., of spoken and written language) and emotional response. Changes in bilateral SMAR gyrus and ipsilateral POPE may be associated with abnormal language processing or a partial displacement of language processing to other regions due to the neuroplastic response to cortical insult, abnormal electrical circuitry, or underlying anatomical anomalies (Devinsky et al., 2000; Hartwigsen et al., 2010).

Changes in specific anatomical regions were associated with disease duration. Notably, the abnormalities of PCUN and LOF which are part of the DMN (Kabbara et al., 2017) strongly related to duration. Prior functional imaging studies showed decreased functional connectivity throughout the DMN and also a relation with disease duration for TLE (Pittau et al., 2012). Epileptic activity was suggested to possibly spread from the temporal lobe into DMN areas (Laufs et al., 2007). The difference in DMN regions compared with controls in our study gets larger with duration (shown in Supplementary Material Table S4), indicating that the DMN might be more severely affected for patients with long‐term TLE. Studies have shown TLE‐related aberrant connectivity was associated with connectivity changes not only to DMN nodes, but also to the DAN (Burianová et al., 2017; Zhang et al., 2009) and the salience network (SAN) (Burianová et al., 2017). This is in accord with our duration‐related and outcome‐related results (Table 2 and Supplementary Material Table S4). For example, PORB and POPE which were located in inferior frontal lobe and associated with DAN showed increasing changed intensity with duration. Especially, the characteristic path length of contra‐lateral PORB was suggested to be a good surgical outcome predictor. Likewise, the INS cortex as parts of SAN, showed large differences in patients and their abnormal intensities get stronger with disease duration. The connectivity strength in the ipsi‐lateral INS network was also stronger for poor surgical outcome patients.

### 4.3 Considerations for surgery

Our analysis of local *within*‐area networks indicated several regions were related to surgery success. Local changes were found to predict epilepsy surgery outcome with an accuracy of about 95.39%. Indeed, the prediction performance is better than the current standard approach of observing global connectivity between regions which has an accuracy of only 88% and better than earlier surgery outcome predictions with 81–88% accuracy (Bonilha et al., 2015; Coan et al., 2016; Sinha et al., 2017). Compared with low‐resolution network studies of *between*‐regions, our high‐resolution predictive method showed better capability with higher AUC (mean: 0.97 vs. 0.95), accuracy (mean: 95.39% vs. 91.45%), and specificity (mean: 100% vs. 88.67%). The predictive power of poor surgery outcome expressed by specificity in our work is crucial since alternative resection targets could be suggested instead of the planned approach. While some of the regions related to bad outcome are also regions relate to a longer disease duration, which in turn makes a bad outcome more likely (such as ipsi‐lateral INS, contra‐lateral LOF, and PORB), other indicated regions are independent of duration. Notably, not all changed regions are facilitating surgery and would therefore be potential surgery targets. Some changes could be due to a relocation of function away from regions affected by seizures that can no longer perform a function. For example, the developing brain can re‐establish language organization in the right hemisphere after left‐side injury (Krägeloh‐Mann, 2004). Indeed, language‐related brain regions have a wider spatial distribution in epilepsy patients (Devinsky et al., 2000). However, the locally changed regions could be good biomarkers for clinical diagnosis before surgery is performed. Making strategies according to the network metric distance to intervene, for example, interrupting ipsi‐lateral INS/PTRI interactions might be promising strategies. Not considering the effects of contralateral regions in current temporal lobectomy may be one reason of surgery failure as our studies demonstrated strong contralateral abnormalities in bad‐outcome patients. The marked regions by up/down arrows in Table 2 and Supplementary Table S5 may provide new directions for future surgery and diagnosis since there are differences in patients with good and poor surgical outcome. Structural network metrics of *within*‐regions may therefore help to inform the planning of surgical interventions in the future.

### 4.4 Brain parcellation and network resolution effect

In our analysis, the DK atlas was used to assign regions to the high‐resolution network. One of the motivations to select the DK atlas is that it has relatively fewer parcellations (68 regions). The computational time therefore less, especially when multiple network metrics for all regions are analyzed. The other reason is that the smallest region had around 100 nodes for a high‐resolution parcellation with about 50,000 nodes. The smallest network size matched the number of nodes at the global scale with 68 cortical regions (Supplementary Material Figure S2). For an atlas with ~1,000 regions, the minimum local network size could be fewer than 10 nodes, which seems not suitable for within‐region network analysis. However, given that the DK atlas is based on the brain's geometric information derived from its cortical model, it is unclear if other types of parcellations, such as biological‐informed parcellations, would perform better. To explore the effect of the different atlas, the “HCP‐MMP” atlas (Glasser et al., 2016) with 360 cortical regions was additionally used to construct a low‐resolution network using the NICARA platform. Note that the “HCP‐MMP” atlas was created from clustering multi‐modal data from the Human Connectome Project (https://humanconnectome.org/). The final prediction results based on the new 360‐node network were shown in Supplementary Material Table S6. Compared to the best predictive power of the original 68‐node network (shown in Supplementary Material Table S5, Accuracy: 91.45±2.43%, Sensitivity: 93.05±2.76%, Specificity: 88.67±4.38%), the performance was quite similar (Accuracy: 89.64±1.94%, Sensitivity: 92.48±3.35%, Specificity: 84.67±3.09%), which indicated a small effect of brain parcellation on the predictive ability of epilepsy surgery outcome by using low‐resolution networks.

While the parcellation scheme does not have a big impact on the results, it remains unclear what the effect of node density on the current results as well. Therefore, two additional node densities were tested to construct the structural connectome and re‐assess results: one consisted of ~25,000 nodes (25 k, average vertex area: ~6.3mm^2^); the other consisted of ~12,500 nodes (12 k, average vertex area: ~12.6mm^2^). The structural connectivity comparison at different resolutions was shown in Supplementary Material Figure S6. Edge density was smaller in the fine‐grained network (50 k, that is, ~50,000 nodes) compared with connectomes at 12 k and 25 k resolution. However, the 50 k networks had a larger characteristic path length, local efficiency, and smaller global efficiency compared with the 12 k‐resolution network which is in agreement with previous studies (Rafael, Mercedes, Line, & Jose, 2012; Zalesky et al., 2010). When looking at the results about duration (Supplementary Material Figure S7), the number of abnormal cortical areas decreased with the Cohen's *d* threshold (i.e., lines decline in the figure, Figure S7(a)). Longer‐duration patients had more changed regions at both 25 k and 12 k network resolution. Besides, they all saw positive relations between alteration intensity and epilepsy duration (Figure S7(b), Pearson's correlation coefficient for 25 k/12 k cases: 0.6672/0.6588; Spearman's rank coefficient for 25 k/12 k: 0.7505/0.7188). The linear fitting lines at three resolutions were almost parallel (Pearson's correlation coefficient for 50 k/25 k/12 k: 0.6652/0.6672/0.6588) which indicated a similar change rate with duration regardless of network resolutions. For patients with more than 20‐year epilepsy, changes also got stronger in the ipsi‐lateral hemisphere at 25 k and 12 k resolution (Figure S7(c), (d)), and regions that changed the most were mostly distributed around ipsi‐lateral PREC and cingulate as well. Similar patterns occurred in outcome‐related results concerning the number of abnormal cortical areas (Supplementary Material Figure S8(a)) and their spatial distribution (Figure S8(b), (c)). As expected, the regional abnormal patterns for the 25 k resolution network as shown in Figure S8(d) were quite similar to the 50 k results shown in Figure 5. The abnormal intensity difference between good‐ and bad‐outcome patients for 12 k and 50 k resolution network was less alike, but there existed some similar regions, such as ipsi‐lateral INS, LING with strong abnormalities in bad‐outcome patients (Figure S8(e)). For the final surgery outcome predictive power (Supplementary Material Table S6), high‐resolution networks were better predictors than low‐resolution networks and among the three high‐resolution networks, while all showing a strong prediction performance, the 50 k‐network was the best.

Overall, both parcellation scheme and node density do introduce some variability in our studies. For the low‐resolution networks, compared to the structural DK atlas, the multi‐modal atlas affects the predictive capacity of epilepsy surgery outcome slightly. While there seems to be no significant difference between the geometric‐ or biological‐informed parcellation for the low‐resolution analysis, the DK atlas with larger intra‐region networks could be better for analysis of connectivity within‐regions. Besides, varying node density from ~50,000 nodes (~3.5 mm^2^) to ~12,500 nodes (~12.6 mm^2^), high‐resolution networks indicate stronger performance than low‐resolution networks. The density of ~50,000 nodes which are constrained by the 68‐node ROIs (DK atlas) would be preferred as the 50 k‐network showed the best prediction and was composed of bigger regions that can benefit other network analyses, such as modularity, in the future.

### 4.5 Methodological limitations and future studies

Concerning limitations of this study, we created structural connectivity only based on cortical regions and while sub‐cortical structures are crucial for seizure propagation, our approach was sufficient to yield biomarkers of epilepsy and of surgery outcome. Secondly, although we found structural network changes, the identification of primary pathology regions should be further examined by other modalities, including EEG and electrocorticography (ECoG) when planning surgical interventions (Lieb, Dasheiff, & Engel, 1991). Thirdly, some region size effects remain even though we eliminated measures that are affected by network size. Although fixing edge density would have been an alternative approach for this challenge in the previous studies (Van Wijk, Stam, & Daffertshofer, 2010), it seems impossible as our high‐resolution network is very sparse and would easily lose local structures if connections were removed to yield a fixed edge density or thresholds across subjects. That is also the reason why we only use the binary high‐resolution structural connectivity without thresholding to get global network properties. To avoid overfitting for the surgery outcome prediction, we used the k‐fold cross‐validation method. However, it also has a big risk of overfitting if the group size is small. More patients should be included in the following analysis to examine results further. Evidence has shown the left TLE patients showed larger differences compared to controls than right TLE (Ahmadi et al., 2009; Besson, Dinkelacker, et al., 2014). To balance the left/right effects, we tried to mitigate this by ensuring the proportion of left and right patients in each duration/outcome group were similar (*p* = 0.335/0.947, Supplementary Table S2). Besides, the group differences were weighted by the sample size of the left and right TLE patients. Nevertheless, the left/right side bias still exists. Future studies would analyze group differences further at the high‐resolution level among left‐/right‐side TLE groups. Finally, we only chose 50,000/25,000/12,500 nodes to generate high‐resolution networks for analysis. Future work may explore more to test which resolutions are most informative for diagnosis and intervention. Besides, the whole high‐resolution network is also informative, studies may also focus on the effect of the whole high‐resolution network on diseases in the next step.

### 4.6 Conclusion

In conclusion, TLE patients show characteristic changes in their structural connectivity *within* regions based on a high‐resolution network. The ipsilateral PREC and the contralateral SMAR gyrus showed large topological changes and some regions, such as the ipsilateral BSTS, PTRI, and contralateral CAC, CMF, LOF, were good predictors of surgery outcome. Specially, ipsilateral INS and contralateral LOF, PORB showed both duration‐related changes and good outcome predictive power. Such areas with local connectivity changes might be candidates for surgery in medically intractable epilepsy. Indeed, *within*‐area connectivity was a better predictor of surgery outcome (mean: 95.39% accuracy) than connectivity changes *between* regions.

## CONFLICT OF INTEREST

The authors declare no competing interests.

## Supporting information


**Appendix** **S1:** Supplementary material

## Data Availability

Data and code availability: The MRI datasets generated during and analyzed during the current study are not publicly available due to data privacy regulations of patient data but high‐resolution connectomes are available upon reasonable request. The massive generation of within region connectivity was handled by NICARA (https://nicara.eu). Network properties were computed by Brain Connectivity Toolbox (https://www.nitrc.org/projects/bct/).
