# Supplementary material for: Connectivity within regions characterizes epilepsy duration and treatment outcome
Source: Hum Brain Mapp. 2021 May 11;42(12):3777–91. doi: 10.1002/hbm.25464 (PMC8288103; doi:10.1002/hbm.25464)
Supplement: Supplementary file 1 — Appendix S1: Supplementary material [file HBM-42-3777-s001.doc]

**Supplementary Information**

Connectivity within regions characterizes epilepsy duration and treatment outcome

Xue Chen^1,2^, Yanjiang Wang^1^, Sebastian J. Kopetzky^3,4^, Markus Butz-Ostendorf^3^, Marcus Kaiser^5,6,2,7^

^1^China University of Petroleum (East China), College of Control Science and Engineering, Qingdao, China; ^2^Newcastle University, School of Computing, Newcastle upon Tyne, UK; ^3^Biomax Informatics AG, Brain Science, Robert-Koch-Str. 2, D-82152 Planegg, Germany; ^4^TUM School of Life Sciences Weihenstephan, Technical University of Munich, Freising, Germany; **^5^**University of Nottingham, NIHR Nottingham Biomedical Research Centre, School of Medicine, Nottingham, UK; ^6^University of Nottingham, Sir Peter Mansfield Imaging Centre, School of Medicine, Nottingham, UK; ^7^Shanghai Jiao Tong University, School of Medicine, Shanghai, China

**Corresponding authors**: Yanjiang Wang; Marcus Kaiser

**Corresponding authors’ e-mail address**:

[yjwang@upc.edu.cn](mailto:yjwang@upc.edu.cn); [**marcus.kaiser@nottingham.ac.uk**](mailto:marcus.kaiser@nottingham.ac.uk)

**Running title**: Within-region structure and TLE treatment

**Supplementary Text S1: Additional information for structural networks and network properties**

Graph theoretical analyses were carried out on low- and high-resolution connectivity networks of patients and controls using the Brain Connectivity Toolbox (Rubinov and Sporns, 2010). We use the term ‘area’ or ‘region’ to refer to a FreeSurfer (DK) area.

The high-resolution networks were constructed based on surface files, the connectivity matrix, ***HM***, was determined by the streamline counts between node pairs and was normalized by node surface area (Besson et al., 2014):

$$HM\left( i,j \right)=\frac{2\sqrt{hsc\left( i,j \right)}}{A_{i}+A_{j}}, i\neq j$$

Where, $HM\left( i,j \right)$ is the connection weight between node $i$ and node $j$ ; $hsc\left( i,j \right)$ records streamline counts between node pairs $i$ and $j$ ; $A_{i}$ is the surface area of node $i$ ; $HM\left( i,j \right)=0$ if $i=j$ . Nodes refer to triangles in high-resolution networks. The connection weights were further scaled by the maximum of $HM$ and defined as $HM$s. We used $S=\sum_{i} \sum_{j} HMs\left( i,j \right)$ to measure **connectivity strength (S)** for both whole high-resolution network (all nodes in the whole brain) and within-area networks (nodes only in a DK area).

The low-resolution connectivity matrix based on DK atlas, ***LM***, was derived from the streamline counts between regions and was normalized by a logistic function (Hutchings et al., 2015):

$$LM\left( i,j \right)=\frac{log(lsc(i,j))}{max(log(lsc))}, i\neq j$$

where $LH(i,j)$ is the connection weight between node $i$ and node $j$; $lsc$ is the matrix of streamline counts; Nodes refer to DK areas in low-resolution network. We calculated $S=\sum_{i} \sum_{j} LM\left( i,j \right)$ to represent **connectivity strength (S)** for whole low-resolution networks and $Si=\sum_{j} LM(i,j)$ to represent the **nodal connectivity strength (Si)** between node $i$ and other regions.

As the high-resolution network is a very sparse network with about 50,000 nodes, we used binary connectivity to calculate the following global network properties. While the low-resolution network is denser with 68 nodes, we used weighted connectivity to be consistent with previous studies.

**Global network properties**

***Edge Density*:** represents the fraction of present connections to the possible connections, which preliminary estimate if network is sparse or dense.

$$d=2E/\left( N\left( N-1 \right) \right)$$

Where $E$ is the number of edges and $N$ is the number of nodes.

***Characteristic path length***: is the average number of edges on the shortest path from one node to another (*Rubinov and Sporns, 2010*).

$$L= \frac{1}{N}\sum_{i} l_{i}=\frac{1}{N}\sum_{i} \frac{\sum_{j\neq i} d_{i,j}}{N-1}$$

Where $l_{i}$ is the average distance between node $i$ and all other nodes, $d_{i,j}$ is the shortest path length between node $i$ and $j$.

***Global efficiency***: As an alternative to characteristic path length, global efficiency is average inverse of shortest path length. Networks with low edge density usually have long path length and are less efficient.

$$E_{global}=\frac{1}{N\left( N-1 \right)}\sum_{i\neq j} d_{i,j}^{-1}$$

***Average clustering coefficient***: measures how well the neighbors of nodes are connected which indicates the average occurrence of clusters.

$$C=\frac{1}{N}\sum_{i} C_{i}=\frac{1}{N}\sum_{i} \frac{2t_{i}}{k_{i}\left( k_{i}-1 \right)}$$

where $C_{i}$ is the nodal clustering coefficient of $i$ ; $t_{i}$ is the number of triangles around node $i$ ; $k_{i}$ the degree of node $i$.

***Average local efficiency***: related to the clustering coefficient, is average global efficiency computed on a subnetwork consisting of neighbors of local node.

$$E_{local}\left( G \right)=\frac{1}{N}\sum_{i} E_{global}\left( G_{i} \right)$$

Where $G_{i}$ is the subnetwork which only consists of the neighbors of node $i$ ; $G$ consists of all nodes in the network.

Characteristic path length, global efficiency, average clustering coefficient and average local efficiency are always sensitive to the sparsity of the network. Due to the low sparsity of high-resolution networks, we normalized with values of 1,000 randomly rewired networks preserving both total number of edges and degree distribution. For comparison, we normalized low-resolution network global properties with values from 100 randomly organized networks with the same total number of edges, degree distribution and strength distribution (*Colon-Perez et al., 2016*).

***Small-worldness***: small-world networks can be characterized by a higher clustering coefficient but a close shortest path length (Watts and Strogatz, 1998; Humphries and Gurney, 2008) than random networks.

$$\sigma=\frac{C/C_{rand}}{L/L_{rand}}$$

Where $C_{rand}$ and $L_{rand}$ are the average clustering coefficient and characteristic path length of random networks which keep both edges and degree distribution as original network for high-resolution network, and keep edges, degree distribution and strength distribution for weighted low-resolution network. A network is small-world if $\sigma\gg1$*.*

**Nodal characteristics**

Three nodal topological characteristics, including nodal connectivity strength (**Si**), nodal efficiency (**Ei**) and clustering coefficient (**Ci**), were used in low-resolution network analysis. The nodal connectivity strength was computed as the sum of the weights of all the connections of one node, which quantifies nodal connected extent to others and was clearly described above. The nodal efficiency, calculated by $E_{global}(Gi)$ where $G_{i}$ is a subnetwork consisting of the neighbors of region $i$, relevant to information flow to other regions. The nodal clustering coefficient as described in average clustering coefficient, measures the local interconnectivity among neighborhood regions of one DK area.

**Reference:**

Besson, P., Lopes, R., Leclerc, X., Derambure, P., Tyvaert, L. Intra-subject reliability of the high-resolution whole-brain structural connectome. NeuroImage, 2014, 102, 283-293.

Colon-Perez, L. M., Couret, M., Triplett, W., Price, C. C., Mareci, T. H.. Small worldness in dense and weighted connectomes. Frontiers in physics, 2016, 4, 14.

Humphries, M. D., Gurney, K.. Network ‘small-world-ness’: a quantitative method for determining canonical network equivalence. PloS one, 2008, 3(4), e0002051.

Hutchings, F., Han, C. E., Keller, S. S., Weber, B., Taylor, P. N., et al.. Predicting surgery targets in temporal lobe epilepsy through structural connectome based simulations. PLoS computational biology, 2015, 11(12), e1004642.

Rubinov, M., Sporns, O.. Complex network measures of brain connectivity: uses and interpretations. Neuroimage, 2010, 52(3), 1059-1069.

Watts, D. J., Strogatz, S. H.. Collective dynamics of ‘small-world’networks. Nature, 1998, 393(6684), 440.


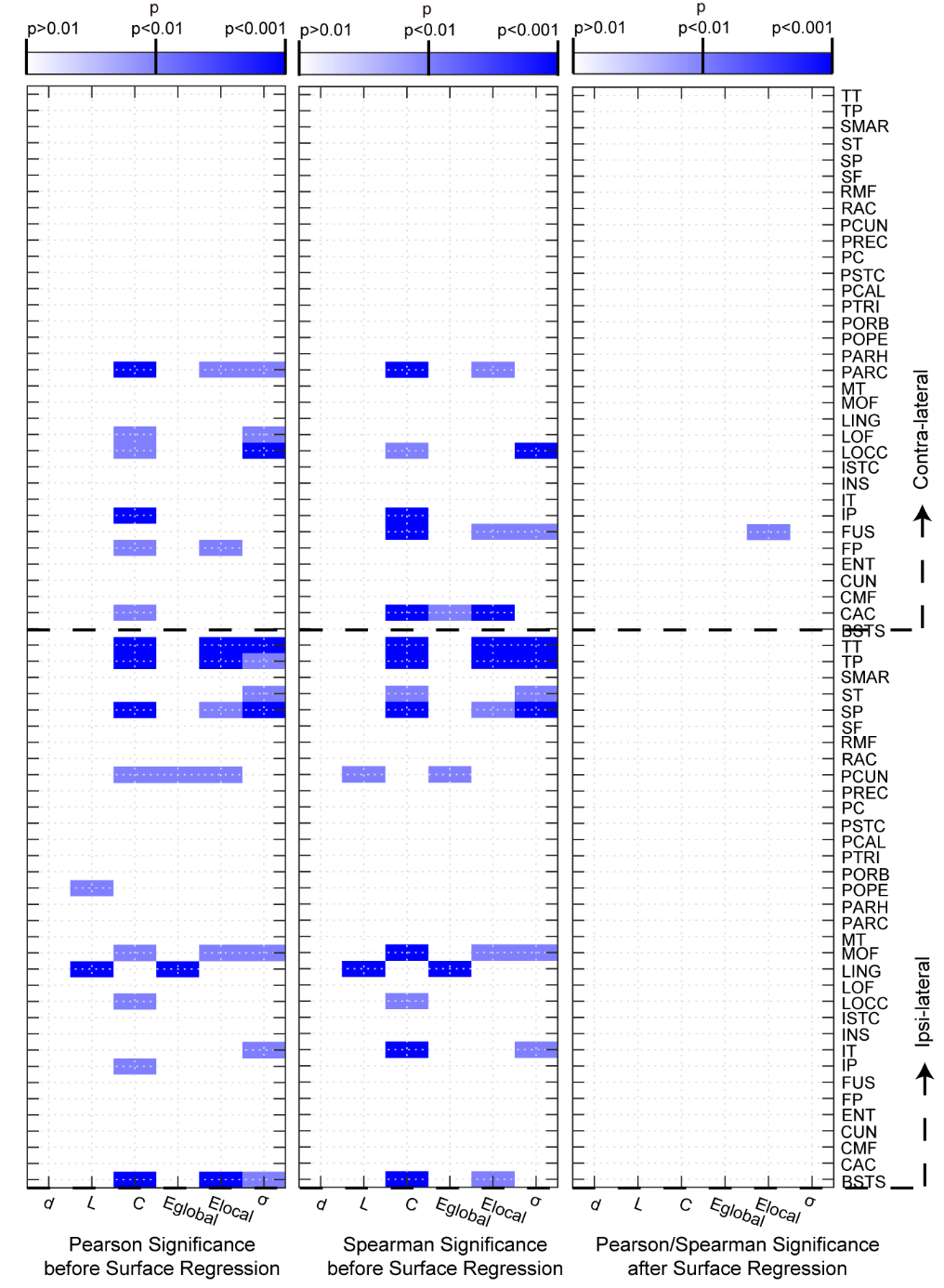


**Supplementary Figure S1 Surface area effect checking before and after it is regressed out.** To assess how surface size changes network properties, we use a general linear model approach. Linear and quadratic effects of surface area are investigated. The model is defined as $network property\sim\beta_{1}*surface+\beta_{2}*surface^{2}+1$, where $\beta_{1}$ and $\beta_{2}$ coefficient represent slope over surface area and quadratic surface area effect, respectively. Two-tailed tests were used and tests were regarded as significant with an $\alpha$ level of 0.05. Pearson/Spearman’s rank correlation is also adapted to describe the linear/nonlinear relationship between surface size and network properties which are derived from cortical local regions (colors were printed when significance level $p<0.01.$ Blue: significant, white: not significant). From left to right: Pearson correlation level before surface regression, Spearman’s rank correlation level before surface regression, Pearson/Spearman’s rank correlation level after surface regression by using the above general linear model. Only one block: local efficiency in contra-lateral fusiform gyrus is shown to be nonlinearly and weakly correlated with surface area ($p=0.009$, $p=0.282$). And it would be eliminated in the following analysis.

**
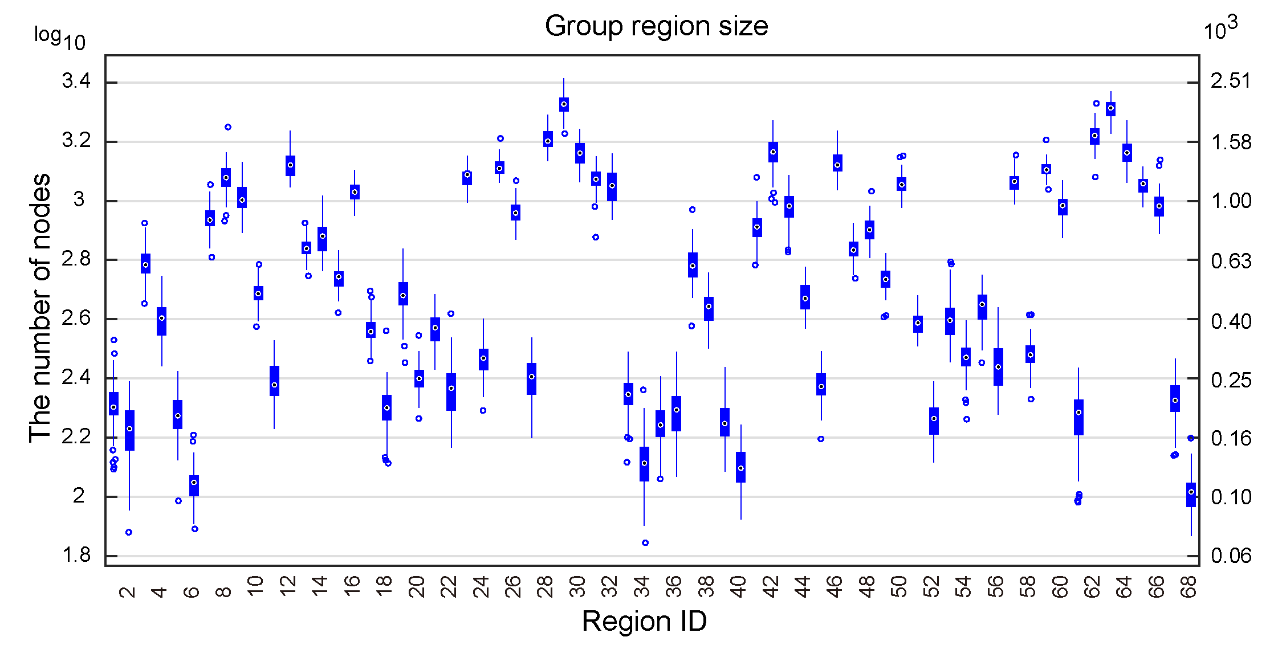
**

**Supplementary Figure S2 The number of nodes for 68 cortical regions.** The X-axis shows the cortical region ID with 1-34 representing left cortical regions and with 35-68 representing right cortical regions. The full region names are listed in Table S1. The Y-axis uses two scales (left: log scale; right: exponential scale) to show the distribution of the number of nodes for each cortical region among all patients and controls. On each box, the central mark indicates the median, and the bottom and top edges of the box indicate the 25th and 75th percentiles, respectively. The whiskers extend to the most extreme data points nor considered outliers, and the outliers are plotted individually using the circle symbol. The smallest region was found with around 100 nodes, whose network size is matched the number of nodes at the global scale with 68 cortical regions.


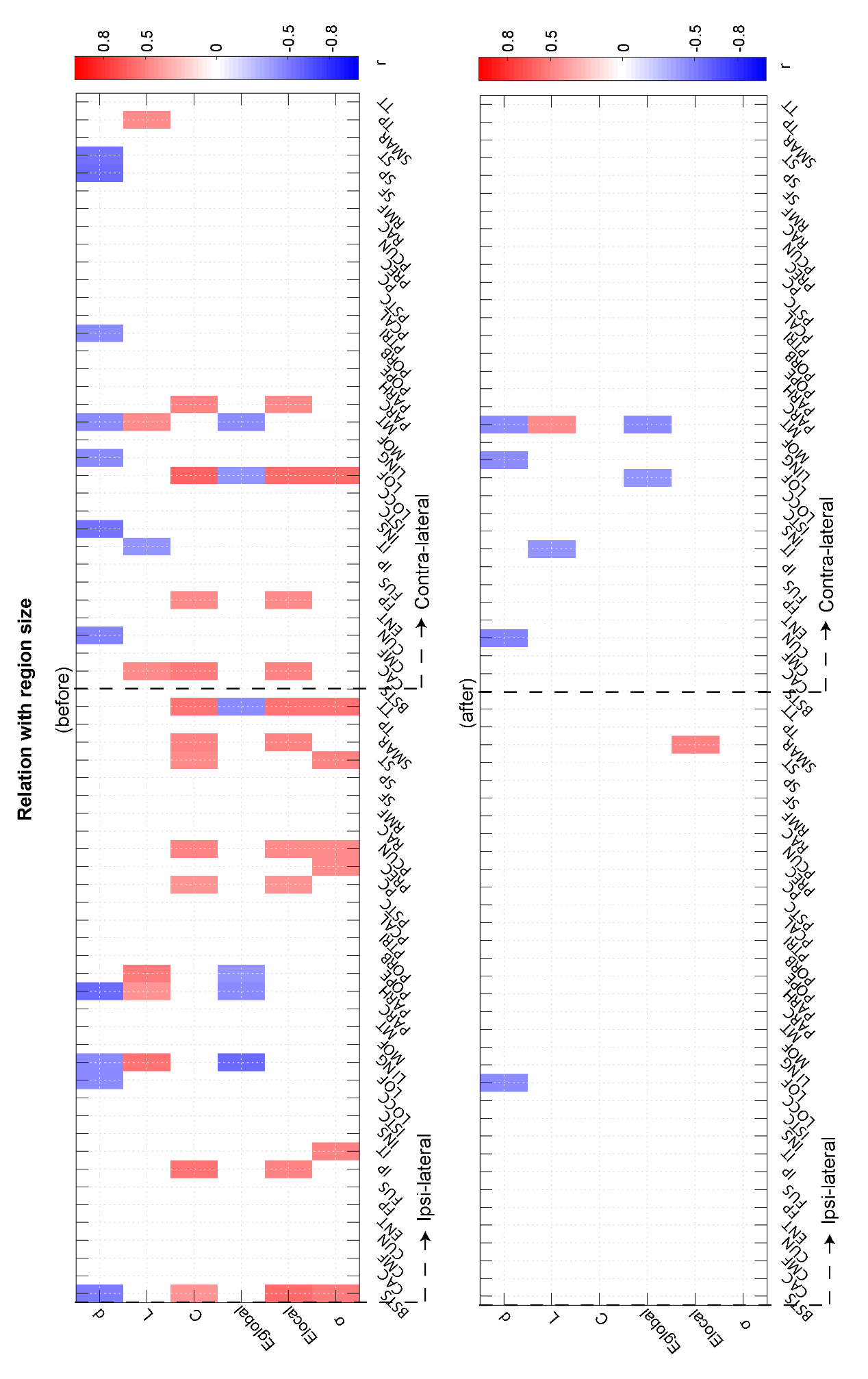


**Supplementary Figure S3 Network size effect after regress out surface area.** Spearman’s rank correlation was adapted to describe surface area effect on network organization measures of cortical local regions (colors were printed only when significance level $p<0.05.$ Red: positive correlation; blue: negative correlation.). As surface area variations does associate with other topology properties, it seems difficult to distinguish whether network structures have big abnormalities or not.To find out the true influence of other metrics in patients, surface area effect was regressed out for each with-area network. Network size (i.e., the number of nodes) is also an effect which should be considered, since it varies across subjects for each DK area covered by similar sized triangles. It has been proved that graph measures, such as normalized clustering coefficient, remain sensitive to network size (especially for number of nodes $N<200$) while keeping the average degree. Such comparisons between networks with different size can therefore yield spurious results. The fact that network measures have no relation with network size in dataset size range will be beneficial for group comparison. To this end, network size effect was checked on metrics of all controls that include various sized within-area networks. The right panel illustrates only few measures in several areas are sensitive to network size after regressing out the effect of surface area, compared with results before regressing out (left panel). In following analysis, we eliminated sensitive parts that were shown on the right panel and only considered measures with small effect of network size.

**
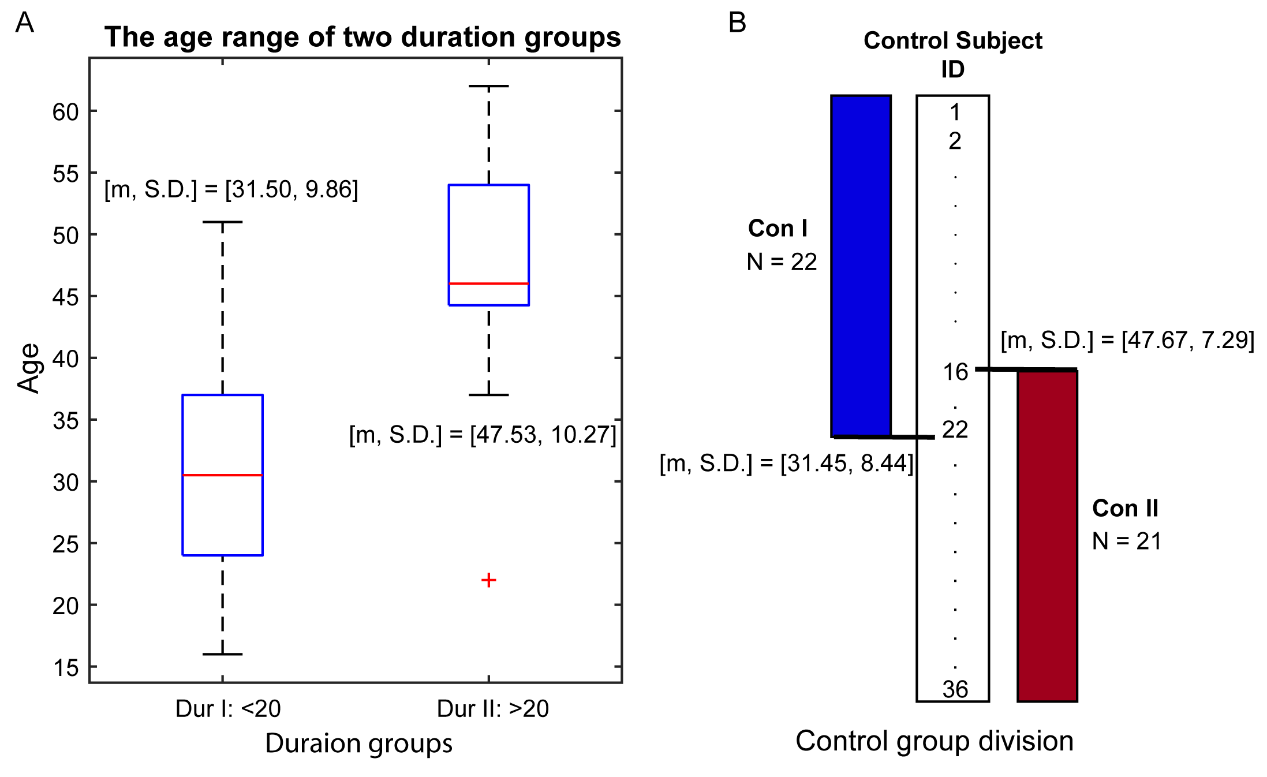
**

**Supplementary Figure S4 Two duration groups and comparable control groups.** (A) Two patient groups were defined according to disease duration: Dur I with duration fewer than 20 years; Dur II with more than 20 years history of epilepsy. The age distribution for each group was described by boxplot. Mean value (m) and standard deviation (S.D.) were listed in the left panel. Significant age difference ($p<0.001$) was found between two groups. (B) For comparison, controls were also categorized into two groups (Con I shown as blue color; Con II shown as red color) whose age and gender were matched with duration groups. Besides, as shown in the right panel, the two control groups were maximum overlapped. The mean value and standard deviation of age were listed as well. There is no age ($p>0.5$) and gender ($p>0.5$) difference between Dur I and Con I (or between Dur II and Con II). See comparison details in Table S2.


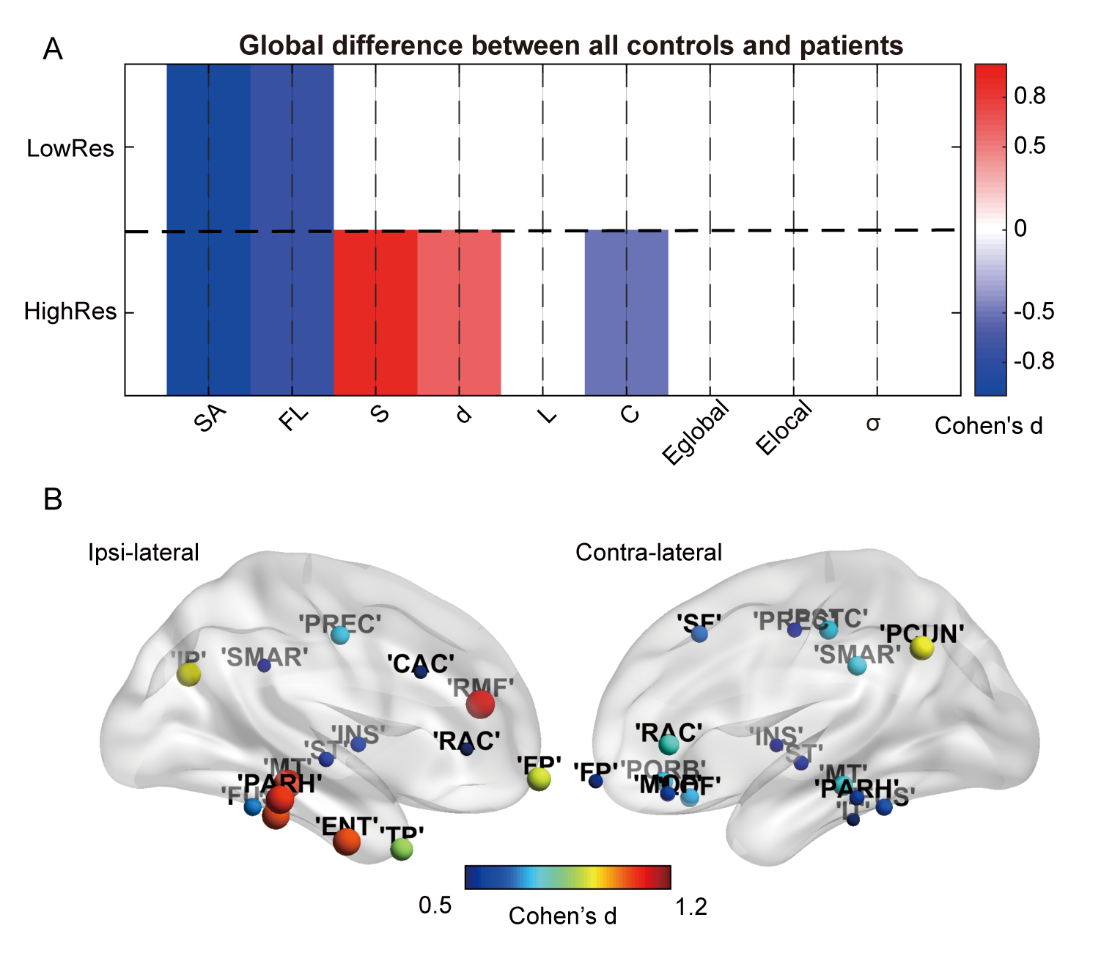


**Supplementary Figure S5** **Patterns of network metrics changes.** (A) The difference of global metrics in whole low- and high-resolution networks. Network metric comparison between controls and patients were performed using 5,000 permutation test after regressing out surface area effect. Colors were printed in the figure when significance level $p<0.05$. Cohen’s d score was used additionally to measure effect size of difference which was mapped on red-blue color bar (Red: positive, significant increase for patients, blue: negative, significant decrease for patients). Abbreviations in figure: SA: total brain surface area; FL: average fiber length; S: connectivity strength; d: edge density; L: Characteristic path length; C: average clustering coefficient; E_global_: global efficiency; E_local_: average local efficiency; σ: small-world-ness; LowRes: low-resolution network with DK cortical area as nodes; HighRes: high-resolution network with triangles on the grey matter surface mesh as nodes. (B) Surface area reduction for regional networks. All comparisons were performed using 5,000 permutation test. The regional surface area reduction power was measured by Cohen’s d score, where large reductions were identified in ipsi-lateral temporal and frontal lobe, especially in entorhinal, fusiform, rostral middle frontal and parahippocampal gyrus. Smaller reductions were recognized in contra-lateral areas. All regions shown in the figure have a clear reduction ($p<0.001$).


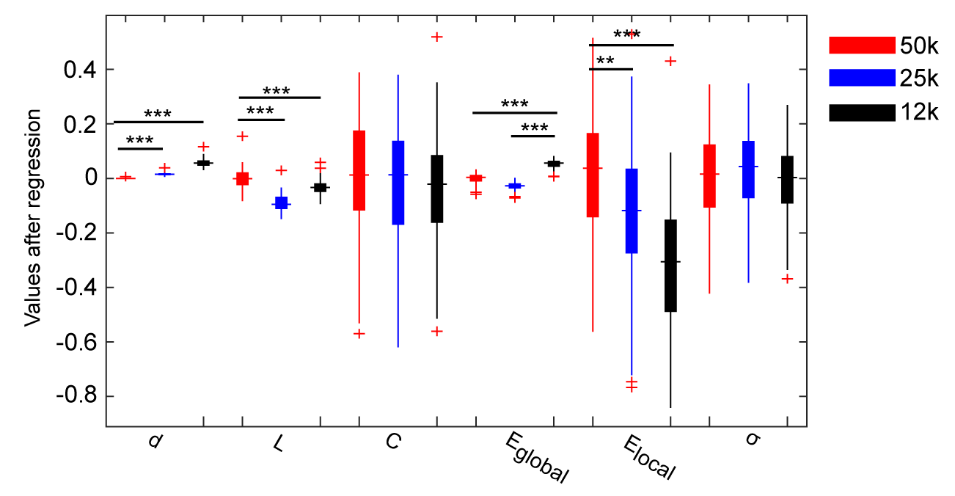


**Supplementary Figure S6** **Network metrics changes with network size.** Same as the pipeline of constructing high-resolution structural connectome with about 50,000 nodes (**50k**) in the main text, another two kinds of structural networks with about 25,000 (**25k**) and 12,500 nodes (**12k**) were built, respectively. Nodes were sorted according to DK atlas. Six topological network properties within regions after regressing out age and gender were studied and compared across networks at different resolutions. Two-sided 5,000 permutation test was used and statistical significance of difference was set: strong (***, p<0.001), medium (**, 0.001<p<0.01), **weak** (*, 0.01<p<0.05). Edge density (d) was shown smaller in a highly grained network (**50k**) compared with that in the connectomes at **12k** and **25k** resolution. Besides, the characteristic path length (L), local efficiency (Elocal) were larger and global efficiency (Eglobal) was smaller in a finer cortical scale (**50k**) compared with that in the **12k**-resolution network, which was in agreement with previous reports (Zalesky et al., 2010; Rafael et al., 2012).

**Reference:**

Zalesky, A., Fornito, A., Harding, I.H., Cocchi, L., et al. Whole-brain anatomical networks: does the choice of nodes matter? NeuroImage 2010,50, 970–983.

Rafael, R. G., Mercedes, A., Line H. C., Jose L.C. Effects of network resolution on topological properties of human neocortex. Neuroimage 2012, 59, 3522-3532.


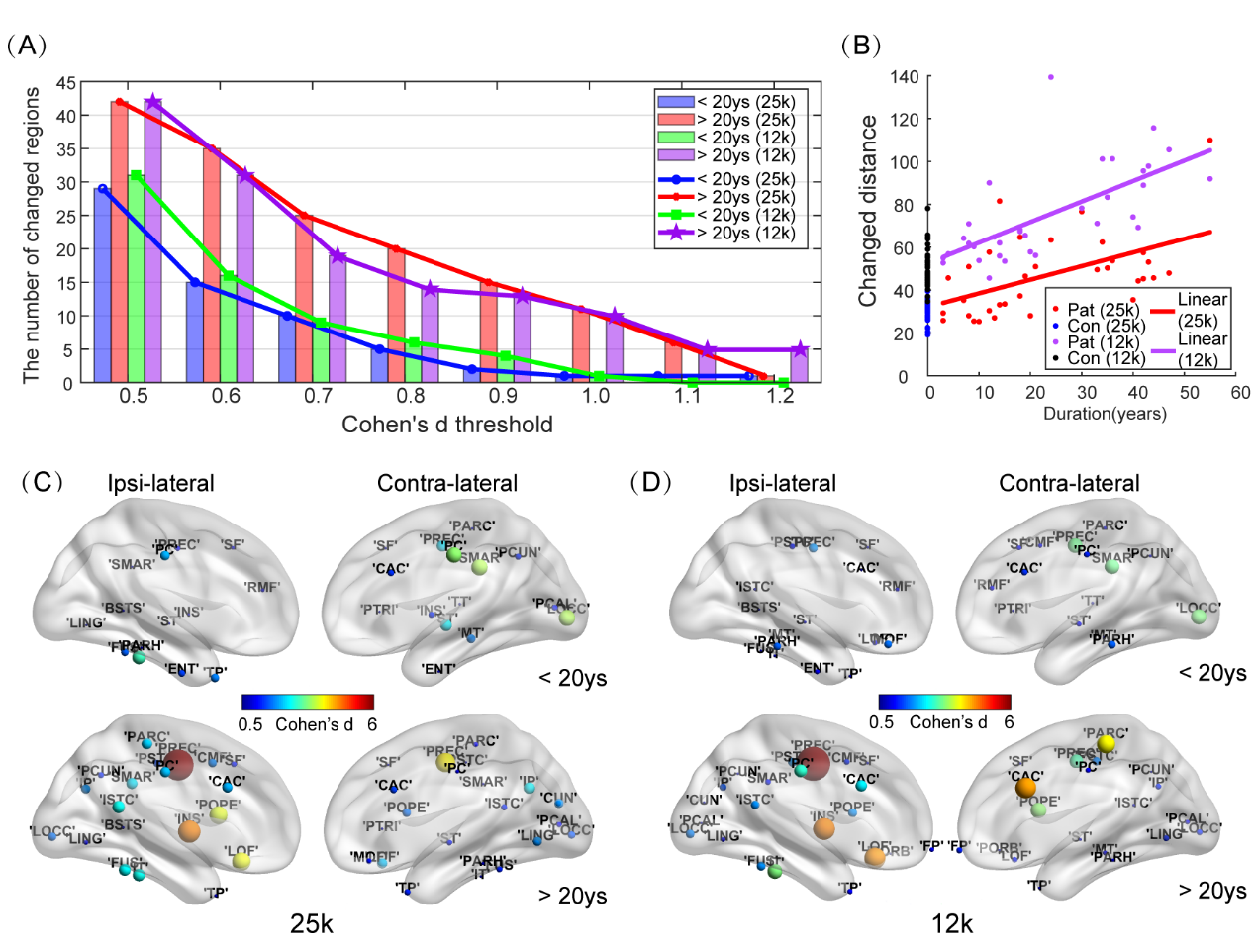


**Supplementary Figure S7** **Intra-regional changes related to the duration at different network sizes.** Patients were also grouped into two parts with a threshold of 20-year disease duration: patients with shorter (<20ys) and longer (>20ys) epilepsy. (A) same as the changed tendency shown in Figure 4 (ref. main text), the number of abnormal cortical areas among 68 regions decreased with the Cohen’s d threshold (i.e., lines decline in the figure). Longer-duration patients (>20ys) had more changed regions compared to the other duration group at both **25k** (about 25,000 nodes) and **12k** (at about 12,500 nodes) network resolution. (B) By summing up z-scores across network features for regions related to duration to give a distance from controls, both **25k-** and **12k-** resolution networks saw positive relations between alteration intensity and epilepsy duration (Pearson’s correlation coefficient for **25k/12k** cases: 0.6672/0.6588; Spearman’s rank coefficient for **25k/12k**: 0.7505/0.7188). Moreover, the two linear fitting lines were almost parallel which indicated a similar rate of change with duration regardless of networks at **25k**, **12k** resolution. (C) and (D) depict changed regions ($p<0.05$, Cohen’s d threshold = 0.5) of two groups at **25k** and **12k** resolution, respectively. Similar to the results at **50k** resolution (about 50,000 nodes) in Figure 4, for patients with more than 20-year epilepsy (in the lower row), changes got stronger in the ipsi-lateral hemisphere (in the left column), and regions that change the most were mostly distributed around ipsi-lateral PREC and cingulate.


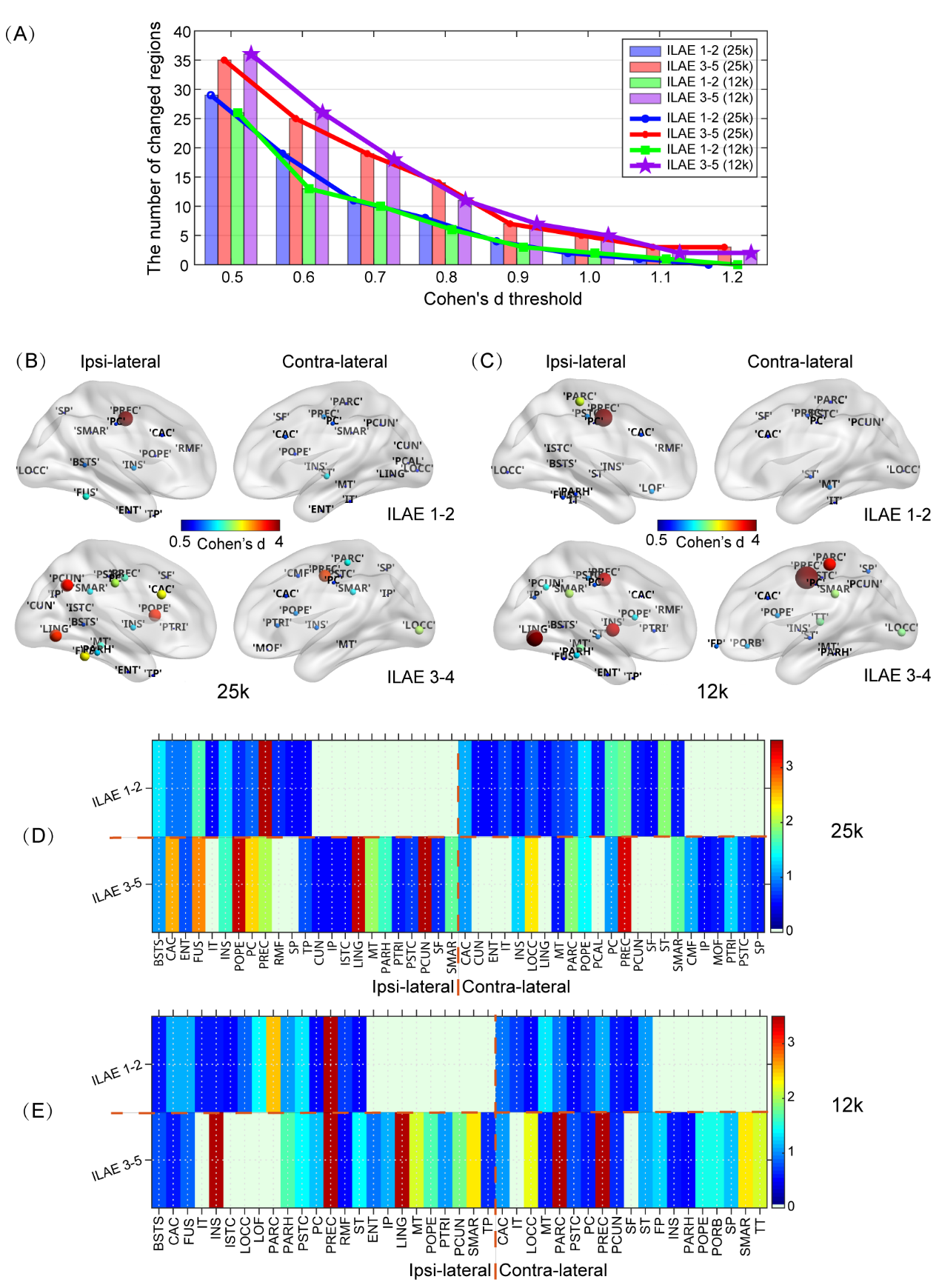


**Supplementary Figure S8** **Local network abnormalities related to the surgical outcome at different network sizes.** Patients were also grouped into two parts: good-outcome patients (ILAE 1-2) and bad-outcome patients (ILAE 3-5). (A) same as the changed tendency shown in Figure 5 (ref. main text), the number of abnormal cortical areas decreased with Cohen’s d threshold (i.e., lines decline in the figure). Patients with bad surgery outcome had more changed regions compared to the other group at both **25k** (about 25,000 nodes) and **12k** (at about 12,500 nodes) network resolution. (B) and (C) showed changed regions ($p<0.05$, Cohen’s d threshold = 0.5) for both outcome groups at **25k** and **12k** resolution, respectively. Similar to the results shown in Figure 5, abnormal regions were also more obvious and widespread in bad-outcome patients (in the lower row). Some regions in the contra-lateral hemisphere, such as the precentral gyrus (PREC) and lateral occipital (LOCC), even had larger abnormalities. (D) and (E) shows the regional abnormality which was estimated by the sum of Cohen’s d scores across all locally changed network features at **25k** and **12k** resolution, respectively. As expected, regional abnormal patterns for **25k** resolution network were quite similar with the **50k** results shown in Figure 5. Some regions such as the ipsi-lateral caudal anterior cingulate (CAC), fusiform (FUS) and insula (INS) were changed stronger for bad-outcome patients. On the contrary, the abnormal intensity difference between good- and bad-outcome patients for **12k** and **50k** resolution network was less alike, but there existed some similar regions, such as ipsi-lateral INS, lingual (LING) with strong abnormalities in bad-outcome patients.

**Table S1: Abbreviations of cortical DK areas**

Areas in the left cortex were ordered from 1 to 34 and in the right were ordered from 35 to 68. The areas in the following are in the left cortex:

1. Banks of the superior temporal sulcus (BSTS);
2. Caudal anterior cingulate (CAC);
3. Caudal middle frontal (CMF);
4. Cuneus (CUN);
5. Entorhinal (ENT);
6. Frontalpole (FP);
7. Fusiform (FUS);
8. Inferior parietal (IP);
9. Inferior temporal (IT);
10. Insula (INS);
11. Isthmus of the cingulate (ISTC);
12. Lateral occipital (LOCC);
13. Lateral orbitofrontal (LOF);
14. Lingual (LING);
15. Medial orbitofrontal (MOF);
16. Middle temporal (MT);
17. Paracentral (PARC);
18. Parahippocampal (PARH);
19. Pars opercularis (POPE);
20. Pars orbitalis (PORB);
21. Pars triangularis (PTRI);
22. Pericalcarine (PCAL);
23. Postcentral (PSTC);
24. Posterior cingulate (PC);
25. Precentral (PREC);
26. Precuneus (PCUN);
27. Rostral anterior cingulate (RAC);
28. Rostral middle frontal (RMF);
29. Superior frontal (SF);
30. Superior parietal (SP);
31. Superior temporal (ST);
32. Supramarginal (SMAR);
33. Temporal pole (TP);
34. Transverse temporal (TT);

**Supplementary Table S2: Duration group and** **surgical outcome group difference.** Patients were separated into two groups according to epilepsy duration: Dur I with duration less than 20-year and Dur II with more than 20-year history of epilepsy. Con I and Con II were age-, sex-matched healthy control groups with Dur I, Dur II, respectively. For surgical outcome, patients were also categorized into two groups according to ILAE outcome scale when compared with all control subjects (Con): Out I corresponds to good outcome patients with ILAE class 1 to class 2; Out II corresponds to bad outcome patients with ILAE class 3 to class 5. The abbreviation m and S.D. represent the mean value and standard deviation of group. Comparison significance was obtained using a chi-square test for gender, surgical outcome, and side of surgery and using a 5,000 permutation test for age, duration. Bonferroni correction was performed due to the multiple comparison.

|  | Num. | Gender  Male/Female | Age  m/S.D. | Duration  m/S.D. | Surgical outcome  ILAE 1-2/ILAE 3-5 | Surgery side  L/R |
| --- | --- | --- | --- | --- | --- | --- |
| Con I | 22 | 12/10 | 31.45/8.44 | -- | -- | -- |
| Dur I | 18 | 8/10 | 31.50/9.86 | 11.50/5.39 | 9/9 | 12/6 |
| Con I vs Dur I | -- | $\chi^{2}=0.404;$ $p=0.525$ | $p=0.985$ | -- | -- | -- |
| Con II | 21 | 9/12 | 47.67/7.29 | -- | -- | -- |
| Dur II | 15 | 7/8 | 47.53/10.27 | 37.8/8.78 | 10/5 | 9/6 |
| Con II vs Dur II | -- | $\chi^{2}=0.051$ $p=0.821$ | $p=0.952$ | -- | -- | -- |
| Dur I vs Dur II | -- | $\chi^{2}=0.016$ $p=0.898$ | $p=0.0004$ | $p=0.0002$ | $\chi^{2}=0.157$ $p=0.692$ | $\chi^{2}=0.930$ $p=0.335$ |
| Con | 36 | 17/19 | 39.06/12.32 | -- | -- | -- |
| Out I | 21 | 10/11 | 38.14/11.33 | 22.29/13.68 | -- | 12/9 |
| Out II | 12 | 5/7 | 39.92/15.49 | 25.5/17.61 | -- | 7/5 |
| Con vs Out I | -- | $\chi^{2}=0.001$ $p=0.977$ | $p=0.773$ | -- | -- | -- |
| Con vs Out II | -- | $\chi^{2}=0.112$ $p=0.738$ | $p=0.844$ | -- | -- | -- |
| Out I vs Out II | -- | $\chi^{2}=0.003$ $p=0.955$ | $p=0.698$ | $p=0.557$ |  | $\chi^{2}=0.004$ $p=0.947$ |

**Supplementary Table S3 Ranking of abnormal areas.** (I) and (C) represent ipsi-lateral and contra-lateral regions, respectively. Areas in bold are located in frontal lobe, whereas areas in bold and italic are regions in temporal lobe. About 50 percent of areas that showed abnormality are in temporal and frontal lobe. Areas were ranked in the table from top to bottom and from left to right. Contra-lateral and ipsi-precentral was found serious changes compared to controls.

| Area | Metrics | Cohen’s d | Area | Metrics | Cohen’s d |
| --- | --- | --- | --- | --- | --- |
| (I)PREC | SA/FL/C/E_local_ | -0.73/0.84/-0.98/-0.82 | (I)IP | SA | -0.93 |
| (C)SMAR | SA/S/C/σ | -0.73/0.72/-0.84/-0.85 | **(I)FP** | SA | -0.91 |
| (C)LOCC | FL/S/d | 0.94/1.30/0.82 | **(I)POPE** | S | 0.90 |
| *(I)FUS* | SA/L/E_global_/E_local_ | -0.95/0.64/-0.63/0.54 | ***(I)TP*** | SA | -0.87 |
| *(I)ENT* | SA/FL/S | -1.06/0.72/-0.64 | (C)PCAL | FL | 0.84 |
| (I)PC | FL/S/d | 0.95/0.61/0.57 | (I)LOCC | FL | 0.79 |
| (I)INS | SA/S/d | -0.60/0.84/0.66 | (C)RAC | SA | -0.78 |
| (C)PORB | SA/C/E_local_ | -0.73/-0.66/-0.62 | (C)CAC | d | 0.78 |
| (I)SMAR | SA/S/σ | -0.52/0.86/-0.58 | **(C)PTRI** | S | 0.78 |
| (C)PREC | SA/FL/E_global_ | -0.56/0.56/0.63 | **(C)SP** | FL | 0.76 |
| (I)ISTC | FL/S | 1.10/0.51 | ***(C)MT*** | SA | -0.76 |
| (C)INS | SA/S/d | -0.52/0.56/0.53 | (I)CUN | FL | 0.72 |
| (C)LING | FL/S | 1.04/0.55 | (C)PSTC | SA | -0.72 |
| (C)PCUN | SA/d | -0.93/0.59 | **(I)SF** | σ | -0.70 |
| (C)LOF | SA/FL | -0.71/0.77 | **(I)PARC** | FL | 0.65 |
| *(C)IT* | SA/FL | -0.50/0.88 | **(C)SF** | SA | -0.65 |
| (I)CAC | SA/d | -0.51/0.83 | ***(C)FUS*** | SA | -0.63 |
| (C)MOF | SA/FL | -0.58/0.63 | ***(I)BSTS*** | d | -0.60 |
| (I)PCUN | S/σ | 0.57/-0.62 | ***(I)ST*** | SA | -0.58 |
| (I)RMF | SA | -1.10 | ***(C)PARH*** | SA | -0.56 |
| *(C)ST* | SA/σ | -0.56/-0.54 | (C)PC | d | 0.56 |
| *(I)MT* | SA | -1.09 | (I)PSTC | L | -0.56 |
| *(I)PARH* | SA | -1.08 | **(C)PARC** | E_global_ | -0.55 |
| *(I)IT* | SA | -1.06 | ***(C)BSTS*** | E_global_ | -0.54 |
| (I)LING | FL | 0.99 | **(C)FP** | SA | -0.53 |
| (C)CUN | FL | 0.94 | (I)RAC | SA | -0.50 |

**Supplementary Table S4 Abnormal areas related to duration.** (I) and (C) represent ipsi-lateral and contra-lateral regions, respectively. Two methods were used to examine if abnormality in specific areas correlate with duration time. Two indices: $r$ (correlation coefficient) and $p$ (statistical significance level) for both Pearson correlation and Spearman’s rank correlation methods measure possible relationship. Abbreviations: DMN—default mode network, DAN—dorsal attentional network, SAN—salience network. Three functional networks are mentioned as some areas are part of resting-state functional networks.

| Area | Pearson | Spearman | Con vs Pat | Network |
| --- | --- | --- | --- | --- |
| (I)CAC | $r=0.4924;$ $p=0.0036$ | $r=0.5210;$ $p=0.0019$ | $d=0.4367;$ $p=0.0656$ | DAN |
| (C)PCAL | $r=0.4967;$ $p=0.0033$ | $r=0.4743;$ $p=0.0053$ | $d=0.4946$; $p=0.0362$ | -- |
| (I)INS | $r=0.4263;$ $p=0.0168$ | $r=0.3714;$ $p=0.0172$ | $d=0.9334;$ $p=0.0002$ | SAN |
| (C)LOF | $r=0.4061;$ $p=0.0190$ | -- | $d=0.9141;$ $p=0.0002$ | DMN |
| (I)PCUN | -- | $r=0.4060;$ $p=0.0191$ | $d=1.0363;$ $p=0.0002$ | DMN |
| (I)POPE | -- | $r=0.3969;$ $p=0.0222$ | $d=1.0117;$ $p=0.0004$ | DAN |
| (C)PORB | $r=0.3785;$ $p=0.0298$ | -- | $d=0.5205$ $p=0.0230$ | DAN |
| (I)FP | $r=0.3717;$ $p=0.0332$ | -- | $d=0.5986;$ $p=0.0100$ | -- |
| (I)PARC | $r=0.3557;$ $p=0.0422$ | -- | $d=1.1114;$ $p=0.0002$ | -- |
| (C)PCUN | $r=-0.3675;$ $p=0.0354$ | -- | $d=0.6831;$ $p=0.0024$ | DMN |

**Supplementary Table S5 Prediction of surgical outcome using surface area and metrics of low-resolution networks.** (I) and (C) represent ipsi-lateral and contra-lateral areas, respectively. Abbreviations: AUC—area under ROC curve, Acc. —accuracy, Sen. – sensitivity, Spec. – specificity, DMN—default mode network, DAN—dorsal attentional network, SAN—salience network. Three functional networks (FN) are mentioned as some areas are part of resting-state functional networks. Predictive model types: Tree—TR, Discriminant—DM, Logistic Regression—LR, Support Vector Machines—SVM, K-Nearest Neighbor—KNN, Ensemble—ENS. The best predictive models were shown in the table. ALL SA/ ALL Low/ ALL Low+SA—means using the summed absolute z-score values of all predictive SA/low-resolution/low-resolution+SA metrics to predict surgical outcome. 50 repetitions were performed to calculate the mean value and standard deviation. The metrics marked with ↓(↑) were significantly smaller (larger) in poor-surgery patients ($p<0.01$) compared with good-surgery patents. Others without marks only show good classification of z-score but slight difference between two surgical outcome groups. 5000 permutation tests were used in predictions. The prediction performance of one metric is significant when $p<0.05$ and metrics with good predictions were shown in the table.

| Area | Metric | AUC | Acc.(%) | Sen.(%) | Spec.(%) | Model | FN |
| --- | --- | --- | --- | --- | --- | --- | --- |
| (I)CAC | SA | 0.73$\pm$0.02 | 76.00$\pm$2.71 | 84.68$\pm$2.78 | 60.81$\pm$5.85 | SVM | DAN |
| (I)CMF | SA | 0.75$\pm$0.02 | 73.33$\pm$1.28 | 76.67$\pm$1.51 | 67.50$\pm$4.73 | SVM | SAN |
| (I)ENT | SA | 0.75$\pm$0.03 | 72.00$\pm$2.05 | 86.11$\pm$3.42 | 55.56$\pm$4.71 | SVM | -- |
| (I)PORB | SA | 0.74$\pm$0.02 | 74.32$\pm$2.34 | 90.73$\pm$3.71 | 45.61$\pm$4.27 | SVM | DAN |
| (I)PREC | SA | 0.82$\pm$0.03 | 81.21$\pm$2.52 | 85.62$\pm$3.66 | 73.50$\pm$4.02 | KNN | -- |
| (I)RMF | SA | 0.75$\pm$0.04 | 77.58$\pm$3.13 | 83.21$\pm$4.44 | 67.71$\pm$7.59 | KNN | SAN |
| (C)CAC | SA | 0.73$\pm$0.04 | 75.83$\pm$2.82 | 83.47$\pm$6.10 | 65.20$\pm$2.58 | ENS | DAN |
| (C)CMF | SA | 0.71$\pm$0.02 | 74.75$\pm$2.10 | 72.87$\pm$2.52 | 78.03$\pm$5.03 | SVM | SAN |
| (C)FUS | SA | 0.76$\pm$0.04 | 72.20$\pm$2.49 | 86.96$\pm$3.15 | 50.64$\pm$7.32 | KNN | -- |
| (C)ISTC | SA | 0.73$\pm$0.02 | 72.38$\pm$2.03 | 77.49$\pm$3.94 | 68.94$\pm$3.80 | ENS | DMN |
| ALL SA | **--** | **0.79$\boldsymbol{\pm}$0.02** | **74.25$\pm$2.60** | **79.50$\boldsymbol{\pm}$3.38** | **65.10$\boldsymbol{\pm}$5.49** | **DM** | -- |
| (I)INS | Ci | 0.74$\pm$0.05 | 75.76$\pm$4.29 | 78.57$\pm$3.37 | 70.83$\pm$5.89 | KNN | SAN |
| (I)CMF | Ci | 0.81$\pm$0.02 | 75.51$\pm$2.24 | 75.79$\pm$3.52 | 75.00$\pm$0.00 | DM | SAN |
| (I)ISTC | Si↓ | 0.74$\pm$0.02 | 73.07$\pm$0.98 | 71.97$\pm$1.54 | 75.00$\pm$0.00 | DM | DMN |
| (I)LING | Si↓ | 0.79$\pm$0.03 | 73.91$\pm$1.84 | 79.63$\pm$2.74 | 63.89$\pm$4.95 | DM | -- |
| (I)PTRI | Ci↑ | 0.77$\pm$0.03 | 77.50$\pm$3.13 | 79.22$\pm$4.88 | 74.49$\pm$2.02 | ENS | DAN |
| (I)SMAR | Ci | 0.72$\pm$0.01 | 73.79$\pm$1.63 | 85.84$\pm$1.37 | 52.70$\pm$3.95 | LR | SAN |
| (I)TP | Ei | 0.80$\pm$0.04 | 77.37$\pm$1.65 | 99.81$\pm$1.36 | 38.10$\pm$4.17 | SVM | -- |
| (C)RMF | Ci | 0.77$\pm$0.04 | 75.28$\pm$3.07 | 76.19$\pm$3.89 | 73.68$\pm$7.49 | ENS | SAN |
| (C)RMF | Ei | 0.76$\pm$0.02 | 81.58$\pm$1.03 | 90.10$\pm$1.62 | 66.67$\pm$0.00 | DM | SAN |
| ALL Low | **--** | **0.93$\boldsymbol{\pm}$0.01** | **81.33$\boldsymbol{\pm}$1.28** | **80.67$\boldsymbol{\pm}$1.14** | **82.50$\boldsymbol{\pm}$2.53** | **DM** | **--** |
| ALL Low+SA | **--** | **0.95$\boldsymbol{\pm}$0.01** | **91.45$\boldsymbol{\pm}$2.43** | **93.05$\boldsymbol{\pm}$2.76** | **88.67$\boldsymbol{\pm}$4.38** | **DM** | **--** |

**Supplementary Table S6. Confusion matrix indicating the performance of the high-resolution network with about 25,000/12,500 nodes and low-resolution network with 360 nodes in predicting surgical outcome.** **HighRes_25k**, **HighRes_12k** (**LowRes_HCP**): the overall prediction power combined with all high-resolution/local predictors (low-resolution/global predictors). The surface area was considered in all methods. **HighRes_25k** and **HighRes_12k** represent the high-resolution methods with about 25,000 and 12,500 nodes globally. **LowRes_HCP** represents the low-resolution method with 360 nodes globally.

| Network | Predicted Outcome | Actual surgical outcome | |
| --- | --- | --- | --- |
|  |  | **Good = 21** | **Bad = 12** |
| HighRes_25k | **Good = 23.50$\boldsymbol{\pm}$0.54**  **Bad = 9.50$\boldsymbol{\pm}$0.54**  **Accuracy = 92.30$\boldsymbol{\pm}$1.53 (%)** | True positive = 20.98$\pm$0.14  False negative = 0.02$\pm$0.14  True positive rate, or sensitivity = 99.90$\pm$0.67 (%)  False negative rate, or miss rate =  0.10$\pm$0.67 (%) | False positive = 2.52$\pm$0.50  True negative = 9.48$\pm$0.50  False positive rate, or fall-out = 21.00$\pm$4.21 (%)  True negative rate, or specificity = 79.00$\pm$4.21 (%) |
| HighRes_12k | **Good = 21.14$\pm$0.45**  **Bad = 11.86$\pm$0.45**  **Accuracy = 93.39$\boldsymbol{\pm}$2.00 (%)** | True positive = 19.98$\pm$0.14  False negative = 1.02$\pm$0.14  True positive rate, or sensitivity = 95.14$\pm$0.67 (%)  False negative rate, or miss rate = 4.86$\pm$0.67 (%) | False positive = 1.16$\pm$0.55  True negative = 10.84$\pm$0.55  False positive rate, or fall-out  = 9.67$\pm$4.57 (%)  True negative rate, or specificity = 90.33$\pm$4.57 (%) |
| LowRes_HCP | **Good = 21.26$\boldsymbol{\pm}$0.92**  **Bad = 11.74$\boldsymbol{\pm}$0.92**  **Accuracy = 89.64$\boldsymbol{\pm}$1.94 (%)** | True positive = 19.42$\pm$0.70  False negative = 1.58$\pm$0.70  True positive rate, or sensitivity = 92.48$\pm$3.35 (%)  False negative rate, or miss rate = 7.53$\pm$3.35 (%) | False positive = 1.84$\pm$0.37  True negative = 10.16$\pm$0.37  False positive rate, or fall-out =15.33$\pm$3.09 (%)  True negative rate, or specificity = 84.67$\pm$3.09 (%) |
